# Vertically distinct microbial communities in the Mariana and Kermadec trenches

**DOI:** 10.1371/journal.pone.0195102

**Published:** 2018-04-05

**Authors:** Logan M. Peoples, Sierra Donaldson, Oladayo Osuntokun, Qing Xia, Alex Nelson, Jessica Blanton, Eric E. Allen, Matthew J. Church, Douglas H. Bartlett

**Affiliations:** 1 Marine Biology Research Division, Scripps Institution of Oceanography, University of California San Diego, La Jolla, CA, United States of America; 2 Department of Soil Science, North Carolina State University, Raleigh, NC, United States of America; 3 Center for Microbial Oceanography: Research and Education, C-MORE Hale, University of Hawaiʻi at Mānoa, Honolulu, HI, United States of America; 4 Flathead Lake Biological Station, University of Montana, Polson, MT, United States of America; Wageningen University, NETHERLANDS

## Abstract

Hadal trenches, oceanic locations deeper than 6,000 m, are thought to have distinct microbial communities compared to those at shallower depths due to high hydrostatic pressures, topographical funneling of organic matter, and biogeographical isolation. Here we evaluate the hypothesis that hadal trenches contain unique microbial biodiversity through analyses of the communities present in the bottom waters of the Kermadec and Mariana trenches. Estimates of microbial protein production indicate active populations under *in situ* hydrostatic pressures and increasing adaptation to pressure with depth. Depth, trench of collection, and size fraction are important drivers of microbial community structure. Many putative hadal bathytypes, such as members related to the *Marinimicrobia*, *Rhodobacteraceae*, *Rhodospirilliceae*, and *Aquibacter*, are similar to members identified in other trenches. Most of the differences between the two trench microbiomes consists of taxa belonging to the *Gammaproteobacteria* whose distributions extend throughout the water column. Growth and survival estimates of representative isolates of these taxa under deep-sea conditions suggest that some members may descend from shallower depths and exist as a potentially inactive fraction of the hadal zone. We conclude that the distinct pelagic communities residing in these two trenches, and perhaps by extension other trenches, reflect both cosmopolitan hadal bathytypes and ubiquitous genera found throughout the water column.

## Introduction

The deep sea is one of the largest biomes on Earth, containing over half of the microbial cells in the ocean [1,2]. Pelagic deep-ocean microbial communities are distinct from those above them [3,4,5,6] and in many cases display higher activities under *in situ* hydrostatic pressures and low temperatures when compared to atmospheric pressure conditions [7]. However, deep-sea environments also contain allochthonous members that descend from above, such as in association with sinking particulate organic matter [8]. These communities can differ from one another, varying by water mass or ocean basin and showing metabolic rates ranging over six orders of magnitude [9]. These variations may reflect resource availability [10,11] and dispersal limitation [12].

More is known about the bathy- and abyssopelagic than the hadopelagic zone, which exists at depths greater than 6,000 m and represents 41% of the oceanic depth continuum [13]. Most hadopelagic sites are associated with trenches, tectonically-active steep-walled depressions that form via subduction. The current study focuses on the Mariana and Kermadec trenches, two hadal sites approximately 6,000 km apart in the Pacific Ocean. The Kermadec Trench in the Southern Hemisphere begins about 120 km off the northeastern coast of New Zealand and reaches its greatest depth at 10,047 m, making it the 5^th^ deepest trench [14]. The Mariana Trench, located in the Northern Hemisphere near the Mariana Islands, extends to 10,984 m at its greatest depth in the Challenger Deep near its southwestern terminus [15], making this the deepest location in the global ocean. Trenches have been proposed to contain unique biodiversity and endemic megafauna due to their geographic isolation [13,16,17,18], but some taxa show a more cosmopolitan distribution, suggesting potential for between-trench dispersal [19,20]. However, studies comparing microbial communities within trenches have not been conducted.

Until recently our understanding of hadal microbial communities has been restricted to highly selective culture-based analyses and small sample size 16S rRNA gene sequence studies [21,22,23]. Distinct microbial taxa adapted to high hydrostatic pressures have been cultured from hadal zones [24,25,26,27,28], of which many are piezophiles, microbes that show optimum growth at pressures greater than 0.1 and as high as 140 Megapascals (MPa; [29]). Recently, increased sample collection and the use of next generation sequencing approaches to hadopelagic communities from the Mariana, Japan, and Puerto Rico trenches have shown that hadal microbial communities are distinct from those above them [30,31,32,33]. At the greatest depths of the Mariana Trench, *Gammaproteobacteria*, including *Pseudomonas* and *Pseudoalteromonas*, are present as major constituents [31,33], findings attributed to trench topography funneling sinking organic matter downward and thereby fueling greater heterotrophic activity [34,35,36,37]. To address microbial community structure, potential endemism, and high-pressure adaptation within hadal trenches, we analyzed the microbial communities inhabiting abyssal and hadal bottom waters within the Kermadec and Mariana trenches using culture-independent high-throughput sequencing, culture-dependent taxonomic characterization, and estimates of microbial activity and abundance.

## Methods

### Sites and sample collection

Kermadec Trench samples were collected aboard the R/V *Thompson* from April to May 2014 using HROV *Nereus* [38], CTD casts, and free-falling/ascending landers (Elevator Lander; [39]). Samples were collected from the Mariana Trench, including within the deepest point of the Sirena Deep [40], aboard the R/V *Falkor* from November to December 2014 using CTD casts and free-falling/ascending landers (Rock Grabber (RG), Schmidt Ocean Institute, https://schmidtocean.org/technology/elevators-landers/; Leggo, Scripps Institution of Oceanography (SIO), https://scripps.ucsd.edu/labs/dbartlett/contact/challenger-deep-cruise-2014/). One sample was also collected from the Challenger Deep in the Mariana Trench. Lander-based seawater samples were recovered from vertically-positioned Niskin bottles 2 m above the sediment-water interface within the benthic boundary layer, and closed ~12 hours after landing to avoid capturing resuspended sediment material. Recovered samples were immediately transferred to a 4°C cold room and their temperature taken. Sampling within the Kermadec Trench was approved under a NIWA Special Permit issued by the New Zealand Ministry for Primary Industries and within the Mariana Trench by the NOAA Monuments office.

### Environmental data

Bathymetry [41] was plotted using the R package marmap [42]. Seawater used for inorganic nutrient analyses was frozen at -20°C and processed at the Oceanographic Data Facility at SIO (S1 Text; https://scripps.ucsd.edu/ships/shipboard-technical-support/odf/documentation/nutrient-analysis). Replicates that varied by over two standard deviations from the mean within each trench were removed. Technical replicates were averaged at each collection site. For cell counts seawater was fixed with 1% paraformaldehyde and stored at -800078C. Samples were later thawed, stained with SYBR Green (Thermo Fisher Scientific, Waltham, MA), and cells enumerated using flow cytometry (Attune Acoustic Focusing Flow Cytometer, Applied Biosystems, Foster City, CA).

### Microbial activity within the Mariana trench

Activity was evaluated using biorthogonal noncanonical amino acid tagging (BONCAT; [43,44]) with the methionine analog homopropargylglycine (HPG; Thermo Fisher Scientific). Seawater was placed into KAPAK bags (Komplete Packaging, Grand Prairie, TX) in 50 mL aliquots in duplicate. Bags were amended with 5 μM HPG, heat sealed, and incubated at 4°C in pressure vessels [45] at 0.1 MPa, *in situ* pressure at collection depth, and 110 MPa to mimic full trench depth. Negative controls were amended with 3% formaldehyde prior to incubation. After 48 hours, samples were fixed with formaldehyde, filtered onto 25 mm, 0.2 μm pore-size GTTP filters (EMD Millipore, Billerica, MA), and stored at -20°C. Active cells were detected as previously described (S1 Text; [43]). The percentage of active cells in each sample was calculated by dividing the number of active cells (DAPI + HPG active) by the total number of cells (DAPI) in each sample. Values from duplicate incubations from each location and pressure condition were averaged and the percent of active cells at one pressure was divided by that at another pressure to determine the effect of pressurization.

### DNA extraction and sequencing

Seawater (40–120 L per sample) was serially filtered through 3.0 (47 mm diameter), 0.2 (47 mm or Sterivex), and 0.1 μm (142 mm) polycarbonate filters using a peristaltic pump. Filters were then placed into a sucrose buffer [46] and frozen at -80°C. DNA was extracted from whole filters using a protocol previously described [33,47]. Negative controls using blank filters were extracted in concomitance with every extraction performed.

The 16S rRNA gene region between 515f-926R was amplified in triplicate for 30 cycles and pooled [48]. Samples were tagged with sample-specific Illumina barcodes during a secondary PCR step, combined at equimolar concentrations, and sent for sequencing on an Illumina Miseq (S1 Text). Overlapping paired reads were merged using FLASH [49] and discarded if they fell below a q score of 33 within a 50 bp sliding window [48] using Trimmomatic [50]. Primers were removed and operational taxonomic units (OTUs) picked at 97% similarity using UCLUST in QIIME 1.9.1 [51]. Chimeras were identified with the Ribosomal Database Project gold database (training database v9) using VSEARCH [52] and removed. Taxonomy was assigned against the SILVA [53] 123 database and sequences identified as contaminants were discarded (S1 Text). Finally, OTUs with fewer than 3 reads in at least 4 samples across the entire dataset were excluded. Sequence data have been submitted to the SRA database under accession numbers SRR5643386-SRR5643480.

### Statistical analyses

Sequencing reads were processed with the R package phyloseq [54]. Samples were rarefied to even sampling depth to account for differing sequencing depths. Alpha diversity was calculated using vegan [55] and comparisons between samples were performed using the beta-diversity metrics Bray-Curtis and weighted Unifrac [56]. Ordinations based on Bray-Curtis dissimilarity and permutational analysis of variance with adonis in vegan were used to identify statistically significant variables. Samples were classified into depth groups of surface, abyssal, or hadal by UPGMA hierarchical clustering using the command *hclust*. Samples were classified based on a hard depth cutoff in each trench; samples that clustered with one depth group but belonged to the other based on depth cutoff were grouped by depth of collection. A differential expression enrichment analysis using DESeq2 [57] was used to test the hypotheses that certain taxa are enriched within specific trenches, hadal zones, and certain size fractions using the un-rarefied dataset [58] with low abundance (at least >1000 total reads per OTU or >3000 reads per phylum) taxa removed. For construction of phylogenetic trees, sequences were aligned using the SINA Aligner [59] and trees built using FastTree [60].

### Isolation and characterization of microbes

Microbes were cultured at 4°C on agar plates at 0.1 MPa or in transfer bulbs (Samco, Thermo Fisher Scientific) at either 0.1 MPa or high pressure. Enrichments from the Kermadec Trench were conducted using 2216 Marine Medium (2216; BD Difco^TM^), A1 Medium, or a seawater minimal medium (S1 Text), while those from the Mariana Trench were conducted in 2216 only. For incubations at high pressure the media was inoculated, mixed with gelatin at a final concentration of 4%, transferred into bulbs, and incubated at the desired pressure [45]. Kermadec Trench samples were incubated at 100 MPa while those from the Mariana Trench were incubated at *in situ* pressure (40–110 MPa). After ~2 months colony forming units (CFUs) were calculated and representative isolates identified via PCR using the primers 27F and 1492R [61].

### Isolate growth and survival

The high-pressure growth and survival characteristics of select strains were evaluated. This included strains from the Mariana Trench isolated at atmospheric pressure; *Pseudomonas* sp. 28, *Pseudoalteromonas* sp. 164, *Psychrobacter* sp. 151, and *Halomonas* sp. 73, and additional strains collected elsewhere; *Pseudomonas pelagia* [62], *Pseudoalteromonas* sp. TW7 [63], *Psychrobacter aquimaris* [64], *Alteromonas mediterranea* [65], and *Alteromonas* sp. SIO [66]. Growth experiments as a function of pressure (0.1, 20, 40, 60, and 90 MPa) and temperature (15°C and 4°C) were set up by inoculating early exponential phase cultures 1:100 into 2216 and either incubated in tubes at 0.1 MPa with shaking at 150 rpm or into bulbs and pressurized. At each time point three bulbs for each strain were sacrificed and their optical density measured (OD600; GENYSIS UV-Vis, Thermo Fisher Scientific).

To estimate survival, early exponential phase cultures were pelleted and reconstituted in 0.2 μm filtered, autoclaved sterile seawater collected from the Scripps Pier. Cultures were then diluted 1:100 into sterile seawater, placed into bulbs, and pressurized at 0.1, 20, 40, 60, and 90 MPa at 4°C. After 30 days, cultures were decompressed and plated on 2216 agar plates at 15°C to estimate surviving CFUs. Cultures were also fixed and total cell counts determined microscopically.

## Results and discussion

### Hadal microbial communities are adapted to high hydrostatic pressure conditions

**Fig 1:**
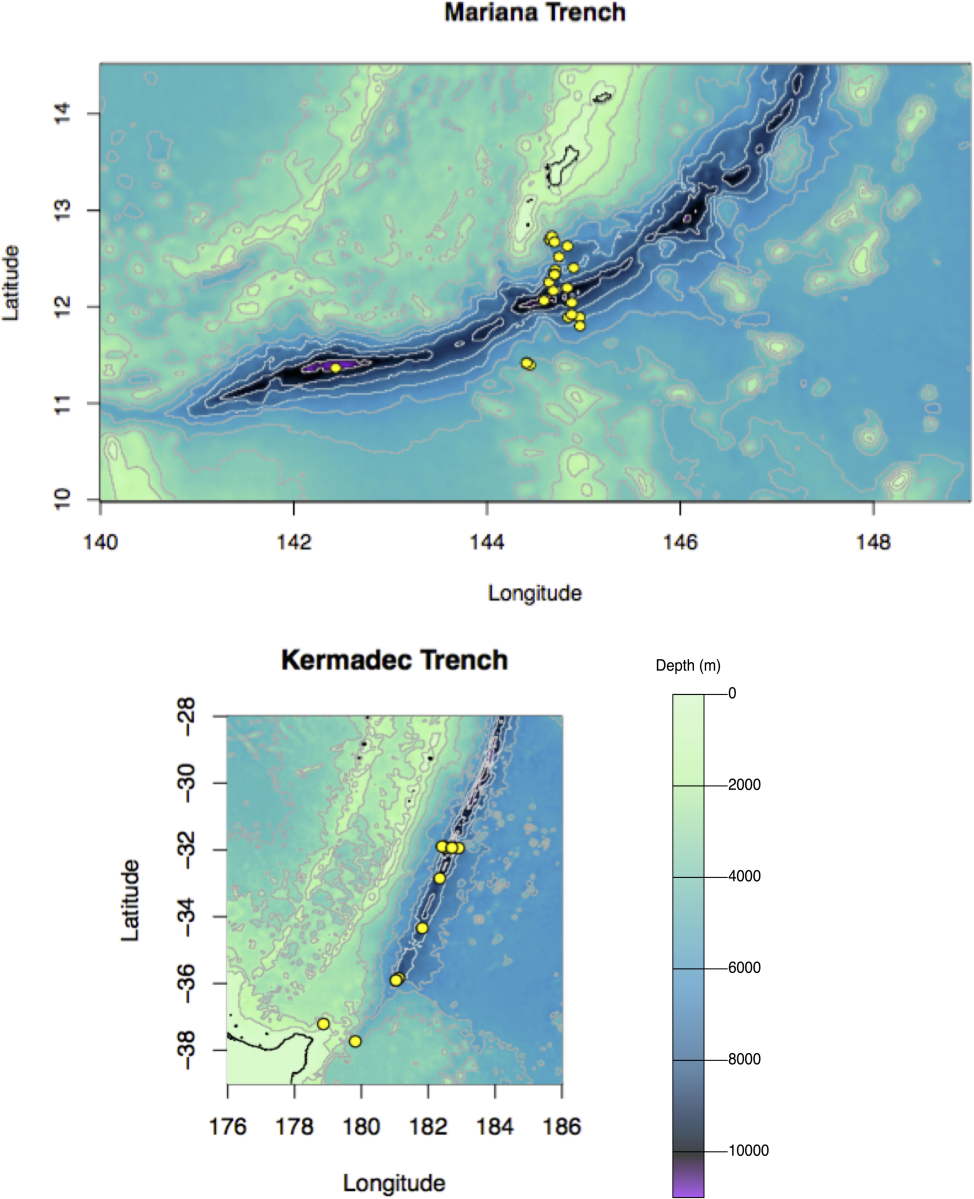
Sample collection locations. Pelagic sample collection locations within the Kermadec and Mariana trenches.

**Fig 2:**
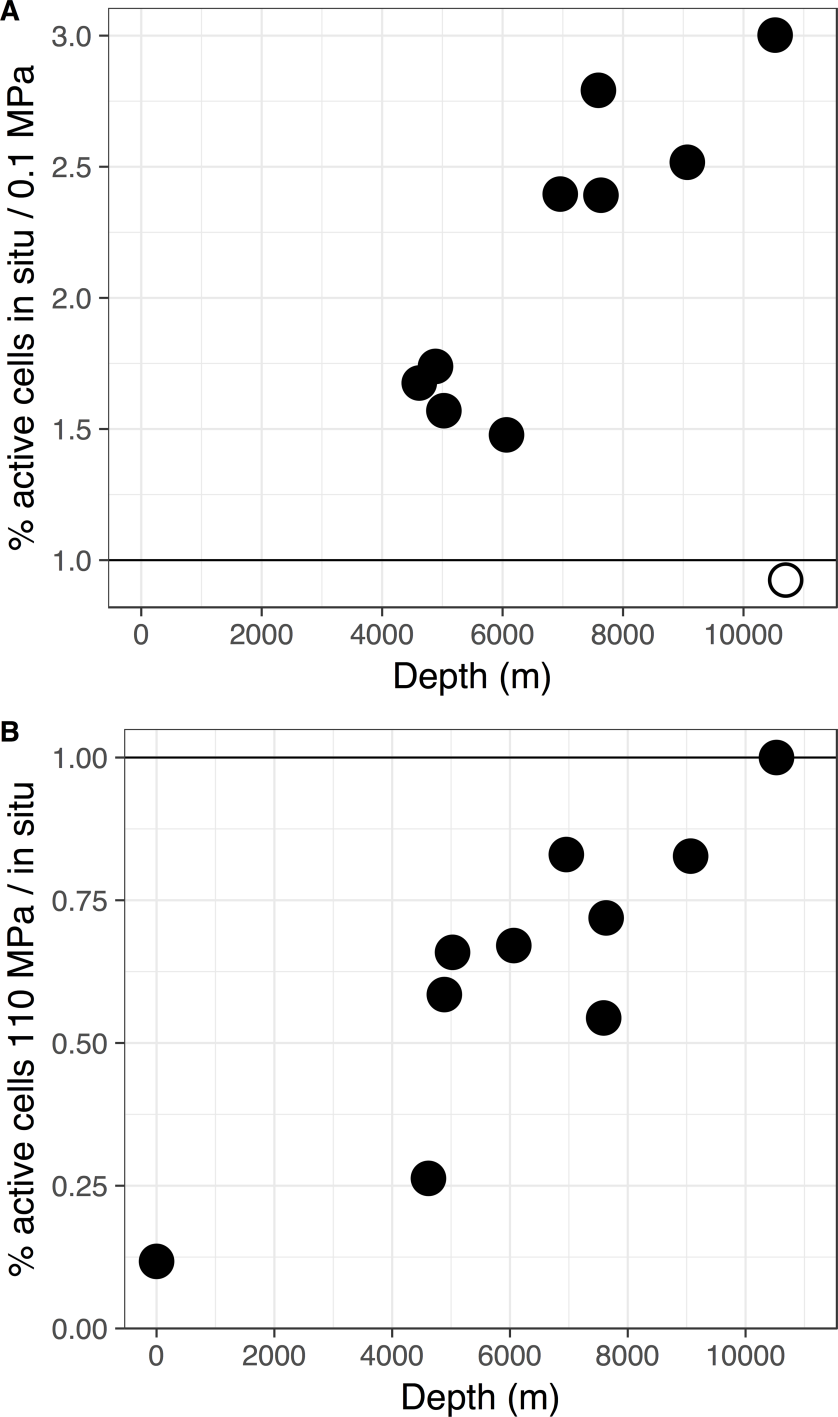
Microbial activity as a function of pressure using HPG. A; Ratio of the percentage of active cells under *in situ* pressure versus atmospheric pressure. B; Ratio of the percentage of active cells at 110 MPa versus *in situ* pressure. Filled circles, seawater collected at <13°C, excluding the surface sample; open circles, seawater collected at >15°C.

Seawater samples were collected at 38 locations within the Kermadec and Mariana trenches at depths up to 10,004 m and 10,920 m, respectively (Fig 1, S1 Fig, S1 Table). Cell abundances of abyssal and hadal samples were approximately 10^4^ cells mL^-1^ (S2 Fig). To assess the activity of microbes collected from the Mariana Trench, the fraction of cells engaged in protein synthesis was evaluated using BONCAT. Although many estimates of deep-ocean microbial activity have been conducted under atmospheric pressure conditions, community activity in stratified waters may be highest under *in situ* hydrostatic pressures [7]. However, such measurements are lacking from hadal locations (e.g. [67,68]). Therefore, the fraction of Mariana Trench cells that were active at atmospheric pressure and following recompression to *in situ* pressures was determined. In the abyssal and hadal samples ~18% (6.5–34.5%) of the cells were active after recompression to *in situ* pressures while ~9% (4.7–13.8%) were active under atmospheric pressure conditions (S3 Fig). Low percentages of active cells may be consistent with prior measurements of microbial activity in the bathypelagic, where activity rates drop over two orders of magnitude from those at the surface and turnover times are estimated to be 0.1–30 years [7,9]. In nearly all samples the proportion of active cells was higher under high hydrostatic pressure (Fig 2). The only exception was a sample from the Challenger Deep, which had warmed to above 15°C during retrieval. These results likely reflect the thermal sensitivity and selective inactivation of autochthonous deep-sea residents over allochthonous microbes from shallower depths [69]. Comparisons of communities incubated at full-ocean depth pressures versus *in situ* pressure showed increasing ratios of full-ocean depth active members with collection depth (Fig 2), indicative of progressive increases in the extent of high pressure adaptation with depth. In contrast to the low percentage of active cells in the deep ocean, over 75% of the community was active in the surface waters at 28°C (S3 Fig). This percentage dropped to ~10% when incubated at 4°C and was further repressed at increasing pressure. We emphasize that bulk community activity was not estimated here, which may exhibit more or less activity as a function of pressure due to the variability of taxa-specific activity rates. Furthermore, benthic boundary layer communities may be more or less active as a function of pressure, relative to the deep pelagic, because these sites are a mixing zone of autochthonous members, allochthonous sinking taxa, and resuspended organic matter from the seafloor [70,71]. Regardless, these findings suggest that hadal communities contain active members adapted to high hydrostatic pressures, even following the stresses imposed by decompression during sample retrieval.

### Hadopelagic communities are unique from abyssal assemblages

**Fig 3:**
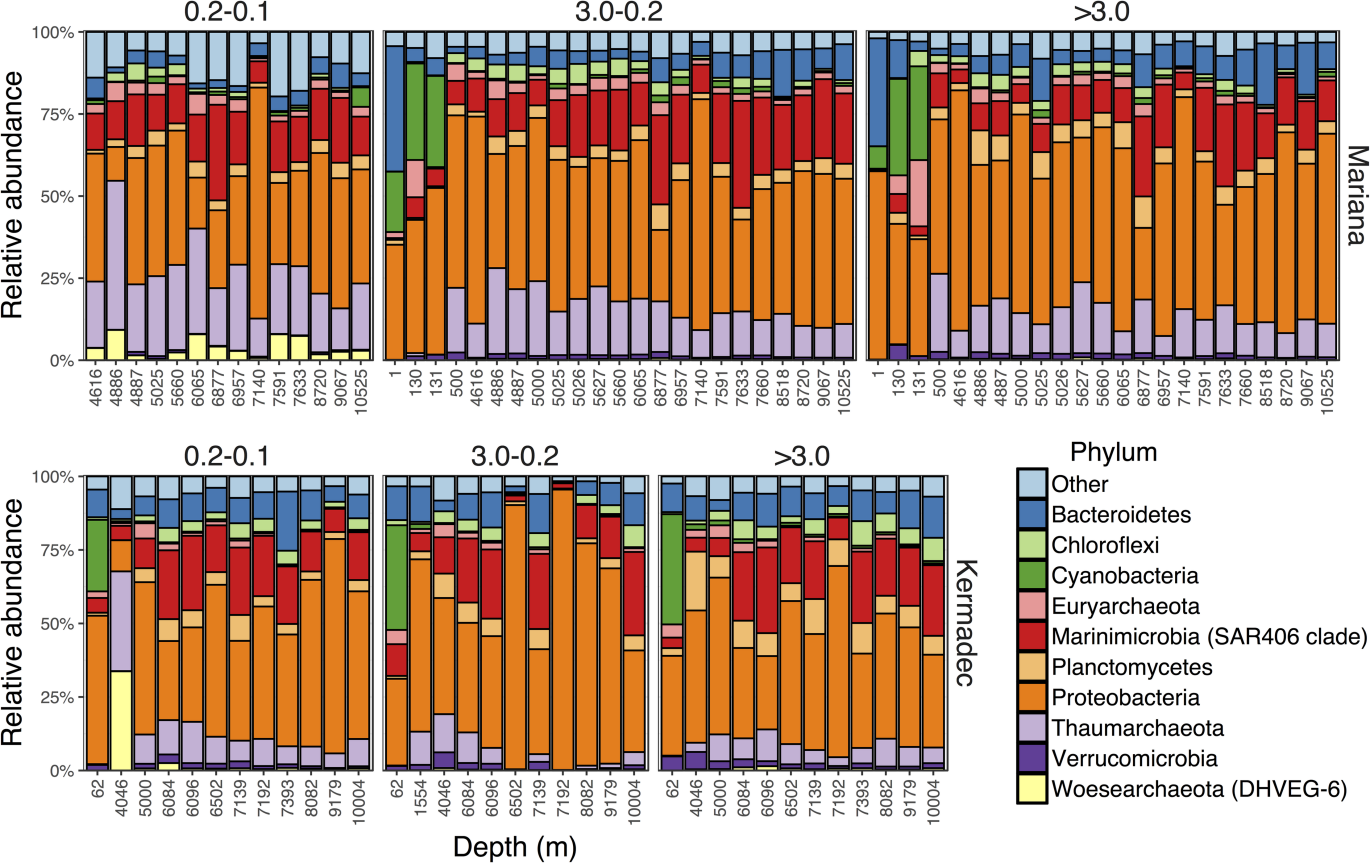
Abundant pelagic phyla. Relative abundances of the ten most abundant phyla in the pelagic zones of the Kermadec and Mariana trenches, organized by trench and size fraction (>3.0, 3.0–0.2, 0.2–0.1 μm).

Microbial communities within the Kermadec and Mariana trenches were compared using high-throughput sequencing of the V4-V5 region of the 16S rRNA gene. The dataset consists of 95 samples and 7,169,109 total sequences, with rarefaction resulting in 15,346 sequences per sample and 8,908 total OTUs (S2 Table). The most abundant group in the abyssal and hadal samples was the *Gammaproteobacteria* within the *Proteobacteria* (Fig 3), composed primarily of *Alteromonas*, *Idiomarina*, *Pseudoalteromonas*, *Psychrobacter*, and *Shewanella*. These taxa represented 10–20% of the community on each size fraction but were sometimes >50%. Other abundant groups included the *Marinimicrobia*, *Thaumarchaeota*, *Bacteroidetes*, and SAR324, consistent with other studies of deep-ocean and hadal communities.

**Fig 4:**
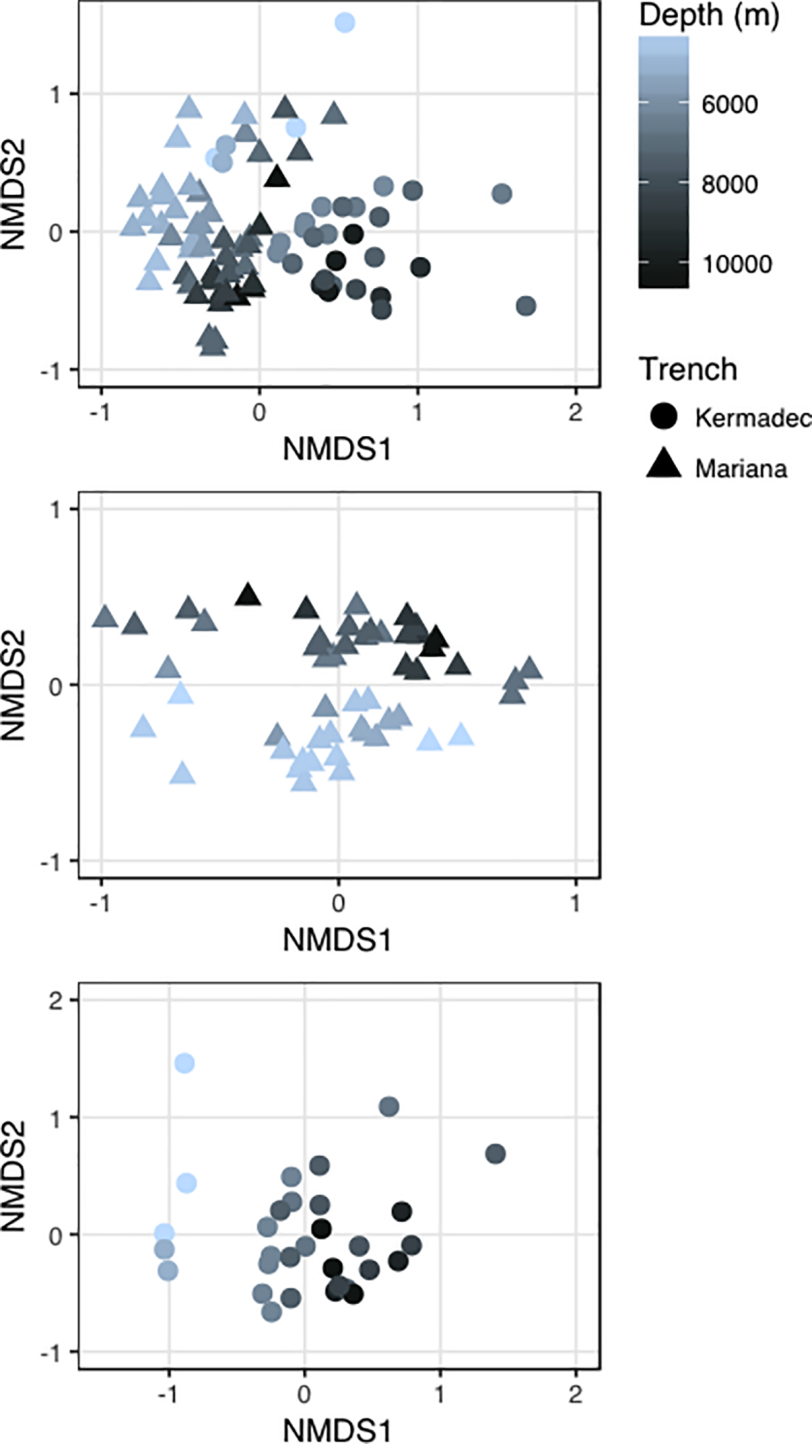
Ordinations of sequenced abyssal and hadal communities. Distances between abyssal and hadal communities visualized via ordinations using Bray-Curtis dissimilarity. A, Mariana Trench; B, Kermadec Trench; C, both trenches.

**Fig 5:**
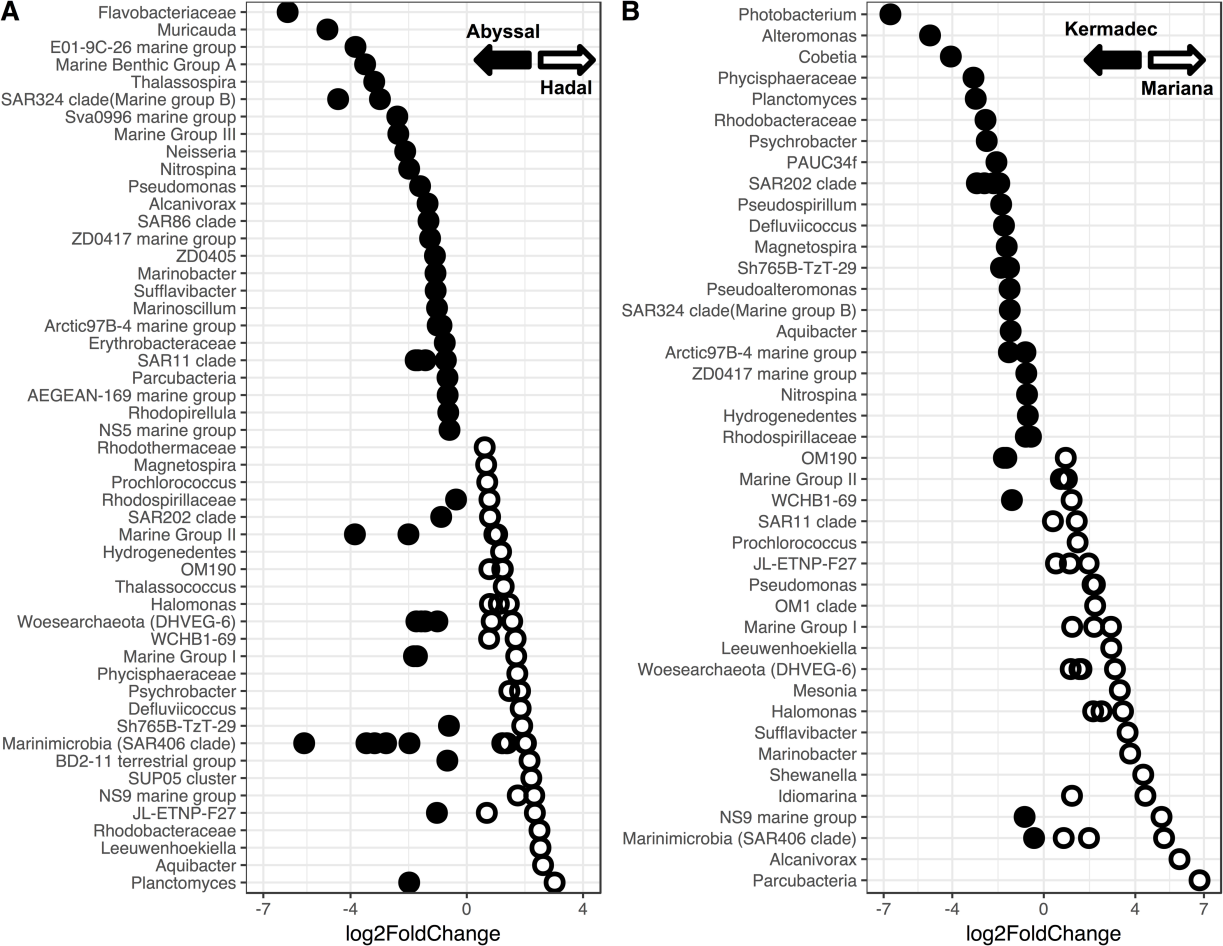
OTUs enriched in the hado- or abysso- pelagic communities. A; OTUs showing enrichment within the hadal or abyssal zones of the Mariana and Kermadec trenches labeled by the lowest discernible taxonomic rank. Filled, enriched in the abyssal zone; open, enriched in the hadal zone. B; OTUs showing enrichment within the hadal zone of the Kermadec or Mariana trench. Filled, enriched in the Kermadec Trench; open, enriched in the Mariana Trench.

Hadal microbial communities have been shown to be different from those present in shallower locales [30,31,32,33]. Here, the hadal communities in the Kermadec and Mariana trenches were distinct from those in the abyssal zone (Fig 4, S4 Fig). Depth was a significant driver of community composition in both trenches (Both trenches, R^2^ = 0.13, p<0.001; Mariana, R^2^ = 0.16, p<0.001; Kermadec R^2^ = 0.21, p<0.001) when comparing samples deeper than 4,000 m. Distinct communities were identified at depths greater than 6,084 m in the Kermadec Trench (Abyssal, 4,046–5,000 m; Hadal, 6,084–10,004 m) and 6,877 m in the Mariana Trench (Abyssal, 4,616–6,065 m; Hadal, 6,877–10,525 m). These findings are similar to changes in megafaunal demersal fish assemblages, which showed distinct hadal communities starting at 6,750 m in the Kermadec Trench and 6,831 m in the Sirena Deep [72]. The *Bacteroidetes* and *Nitrospira* were more abundant in hadal zones and *Thaumarchaeota* and *Chloroflexi* were more abundant in abyssal zones (S5 Fig), which may in part be driven by ammonia concentration/flux and higher amounts of organic matter with increasing depth [31,32,73,74]. Many hadal-enriched taxa identified here (Fig 5, S3 Table) shared sequence similarity with microbes previously obtained from trenches, including OTUs belonging to the *Marinimicrobia*, *Planctomycetaceae*, *Rhodobacteraceae*, and *Flavobacteriaceae*. The *Marinimicrobia* have been observed as one of the most abundant phyla within the Puerto Rico [75] and Mariana [31,33] trenches, consistent with their high abundances seen here. OTUs related to *Aquibacter* (*Flavobacteriaceae*; 96% similar to *A*. *zeaxanthinifaciens*) and *Defluviicoccus* (*Rhodospirilliceae*; 85% similar to *D*. *vanus*) were specifically enriched in the hadal samples and reached abundances of up to 11% and 1.5%, respectively. These OTUs showed high similarity to sequences previously obtained from the pelagic Puerto Rico, Mariana, and Japan trench datasets as well as sediments from the Ogasawara Trench (S6 Fig; [76,77]). These groups may represent bathytypes, taxa adapted to the specific ecological niches associated with depth. Heterotrophic *Gammaproteobacteria*, including *Psychrobacter* and *Halomonas*, were also preferentially enriched within the hadal versus abyssal depths examined, but were found to have widespread, cosmopolitan distributions that include shallow-water locations (e.g. S13 Fig).

### Kermadec and Mariana trench communities are distinct from each other

Trenches may represent independent zoographic provinces [16,17], each with its own unique signature of biodiversity and endemism due to their extreme depth, topography, nutrient inputs, and in many cases isolation from other trench systems. Comparisons of the Kermadec and Mariana trench communities revealed distinctive memberships in each trench (Fig 4, S4 Fig; R^2^ = 0.15, p<0.001). When excluding surface samples, 50% of all OTUs were specific to one trench but made up only 5% of the total sequences. Of these unique OTUs 75% belonged to the Mariana Trench, in agreement with increased richness in the Mariana relative to the Kermadec (S7 Fig). When comparing the abyssal and hadal zones 48% of the OTUs were zone-specific but made up only 5% of all sequences, while 13% of all OTUs were found in both trenches and both pelagic zones and accounted for 83% of all sequences. Therefore, these assemblages contain widespread, abundant lineages and a number of rare, potentially endemic taxa that make up a large number of OTUs but a small fraction of the entire community. Similar findings have been reported for the meso- and bathypelagic [12,78], where these communities are composed of both abundant and widely distributed species as well as rare, sample-specific taxa that represent a minor fraction of the total community [4]. The importance of such low-abundance taxa is not well understood, but they may represent a microbial seed bank capable of becoming more abundant under specific environmental conditions [79].

Taxa were identified that showed differential abundances between the two hadal zones (Fig 5, S4 Table). Regardless of abundance, however, they were found in both trenches and showed similarity to other deep-ocean sequences (e.g. S7 Fig). Therefore these taxa are widespread but differentially abundant within the deep-sea communities. Variations in abundance of depth-cosmopolitan taxa, especially members of the *Gammaproteobacteria*, were also seen. While the Kermadec Trench was enriched in sequences related to *Alteromonas*, *Pseudoalteromonas*, *Photobacterium*, *Cobetia*, and *Psychrobacter*, the Mariana Trench was enriched in sequences related to *Shewanella*, *Alcanivorax*, *Idiomarina*, *Marinobacter*, *Halomonas*, and *Pseudomonas*. All of these sequences show similarity to those within surface waters. The differences in community composition between these two trenches may be due to fluxes of organic matter, as annual rates of primary production in the overlying waters of the Kermadec have been estimated as 87 g C m^-2^ yr^-1^, compared to lower productivity (59 g C m^-2^ yr^-1^) in the waters above the Mariana Trench [13,80]. Water masses, which can have distinct biogeochemical properties, may also affect community composition [81]. Differences in nutrients between the two trenches (S2 Fig) indicate different water mass inputs, with the Kermadec being the first trench in the Pacific Ocean to receive Lower Circumpolar Deep Water [16] while the Mariana receives both Lower Circumpolar Deep Water and North Pacific Deep Water [82]. Site-to-site variations may also be driven by benthic boundary layer nutrient concentrations, perhaps because of differing influences from topographic focusing of settling organic material and/or sediment resuspension. For example, within the Mariana Trench higher relative abundances of nitrogen-cycling *Nitrospira* and *Nitrosomonas* correlated with sites containing higher nitrite concentrations (S8 Fig).

### Community composition differs between size fractions

Different size fractions may represent distinct niches, with larger size fractions containing particle-attached microbes and smaller size fractions including free-living microbes. We separated the trench microbial communities into >3.0 μm (particle-associated), 3.0–0.2 μm (free-living), and 0.2–0.1 μm (ultra-small free-living) size fractions. Size fraction was a small driver of community composition (both trenches; R^2^ = 0.05, p<0.001; Mariana, R^2^ = 0.12, p<0.001; Kermadec, R^2^ = 0.08, p>0.08). The most well represented taxa associated with the particle fraction included members of the phylum *Planctomycetes* (S9 Fig), which have a particle-attached lifestyle in bathypelagic settings [83]. The taxa most enriched in the free-living fraction included *Alteromonas*, SAR324, SAR202, and the *Marinimicrobia*. Comparisons between the free-living and ultra-small free-living size fractions showed that the ultra-small free-living fraction was enriched in three archaeal phyla, including the *Thaumarchaeota*, Marine Hydrothermal Vent Group (MHVG), and *Woesearchaeota*, and two members of the candidate phyla radiation [84], the *Parcubacteria* and *Gracilibacteria* (S10 Fig). Many of these taxa were specifically enriched in the Mariana Trench (Fig 5, S4 Table) and show best, albeit low, similarity to sequences from the Mariana, Japan, and Ogasawara trenches, highlighting their potential uniqueness. The *Parcubacteria* and other members of the candidate phyla radiation represent some of the smallest bacteria known [85]. Our findings support the observation that unique taxa can be exceptionally small and the abundances of many microbes may be misinterpreted when sampling stops at the 0.2 μm pore size.

### Culturable microbes are widespread

Piezophiles have been isolated from a variety of deep-ocean trenches, with most belonging to the genera *Colwellia*, *Shewanella*, *Moritella*, or *Psychromonas* [24,25,26,86,87], r-strategists that grow relatively rapidly in nutrient-rich media. Abundance estimates of these cultured piezophiles indicate that they make up a small fraction of hadal seawater communities despite their consistent isolation [30]. Previously no discernible portion of the community in the Sirena Deep was attributed to known piezophiles, although they made up ~0.5% of the communities in the Challenger Deep [33]. However, distribution and rates of isolation of piezophiles have not been thoroughly investigated.

**Fig 6:**
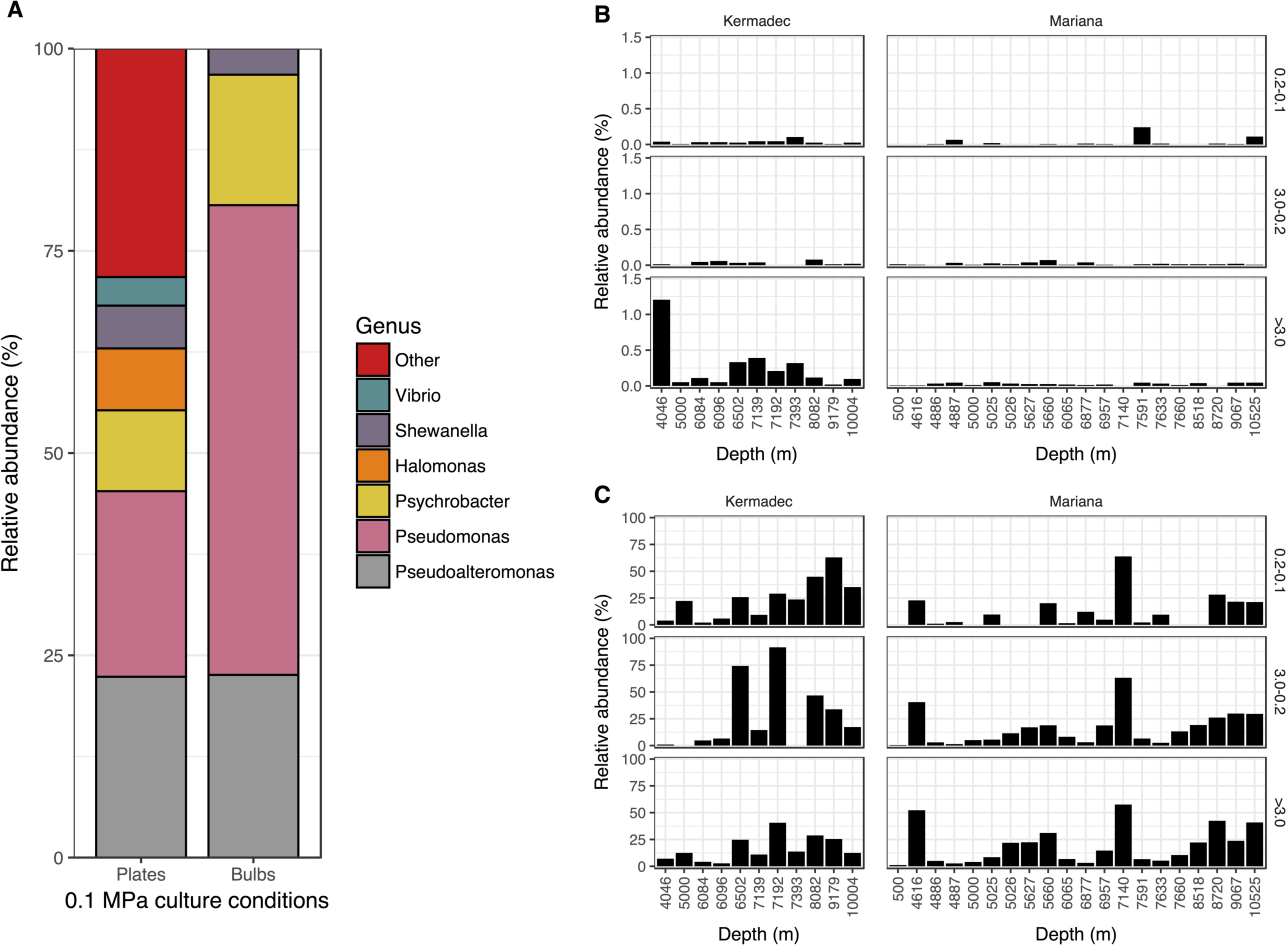
Isolates and their abundances. A; Relative abundances of cultured isolates from seawater in the Kermadec and Mariana trenches at 0.1 MPa and 4°C on plates (n = 170) or in bulbs (n = 31). B; Combined relative abundances of OTUs similar to piezophilic *Colwellia*, *Shewanella*, *Moritella*, or *Psychromonas* species derived from the community data. C; Combined relative abundances of OTUs related to the genera *Pseudoalteromonas*, *Pseudomonas*, *Psychrobacter*, *Halomonas*, *Shewanella*, and *Vibrio* derived from the community data.

To determine if previously isolated piezophilic species were present in the Mariana and Kermadec trenches, the community sequence data was searched for OTUs with >97% similarity to known piezophiles. Abundances were typically less than 1% within each size fraction and were higher in the Kermadec relative to the Mariana Trench (Fig 6; T-test, p<0.04). Sequences related to piezophilic taxa were preferentially enriched on the particle fraction, indicative of a surface-attached lifestyle associated with the utilization of particulate organic matter (T-test, both trenches, p>0.05; Kermadec Trench, p<0.05). Now three trenches, including the Puerto Rico, Mariana, and Kermadec, have been shown to contain sequences associated with previously isolated and cultured piezophiles at relative abundances of less than 1%. Thus, these piezophiles, along with other putative bathytypes, appear to maintain connectivity between trenches, including within the northern and southern hemispheres and the Pacific and Atlantic oceans. Some obligate piezophiles show growth and activity at pressures as low as 40 MPa, suggesting they can survive at abyssal depths. Therefore many hadal microbes, as with other deep-sea microbes [12], could be transported into other regions of the global ocean in association with water mass circulation at abyssal depths. Curiously, no isolates were obtained at high hydrostatic pressure from the water column in either trench (S5 Table). The inability to culture piezophiles here may be due to low abundances of these taxa, especially in the Mariana Trench, or the small numbers of enrichments performed. Because many piezophilic microbes have been isolated from hosts and sediments [24,26,86,88], they may be more abundant in these niches than in the water column.

Many microbes have also been isolated from deep-ocean samples when incubated at atmospheric pressure, but the role of these isolates *in situ* remains unclear. In contrast to our high pressure culturing results, 10^2^–10^3^ CFUs mL^-1^ were obtained from trench samples on plates and in bulbs incubated at atmospheric pressure (S5 Table). Most isolates were related to the genera *Pseudoalteromonas*, *Pseudomonas*, *Shewanella*, *Halomonas*, and *Psychrobacter* (Fig 6, S6 Table). Related taxa have been isolated from other deep-ocean samples [31,89,90,91], suggesting there is selection even for microbes that grow at atmospheric pressure. Interestingly, atmospheric pressure isolates are more representative of deep-ocean communities than microbes isolated under *in situ* conditions (Fig 6). To assess the ability of deep-sea associated, atmospheric pressure-isolated microbes to grow under deep-sea conditions, growth studies were performed for nine isolates belonging to the genera *Pseudoalteromonas*, *Pseudomonas*, *Psychrobacter*, *Alteromonas*, and *Halomonas*, including five deep-sea isolates and four related shallow-water species (S7 Table). None of the isolates were piezophilic or grew at pressures greater than 40 MPa (S11 Fig), consistent with other estimates of high-pressure growth of related strains [89,92,93]. Although growth rates were repressed under the low oxygen conditions that develop in batch high-pressure cultures, the reduced oxygen availability does not explain the pressure sensitivity of these strains. After pressurization for one month at 90 MPa cells remained intact and some strains remained cultivable (S12 Fig), indicating they may remain viable in the deep sea for long periods. Sequences representative of these genera were found in the surface, abyssal, and hadal communities in high abundances (S8 Table, S13 Fig), indicating they may not be obligate bathytypes, and related taxa have been found associated with sinking particles [94,95,96]. Therefore these atmospheric-pressure adapted “deep-sea” isolates could represent cell types which colonize particles, either at the surface or at meso- or bathy- pelagic depths [12,97] and descend to full-ocean depths where they can survive prolonged periods at high pressure. Future studies should evaluate whether the hadal populations of these genera represent a mixture of closely-related strains that show ecotype differentiation (e.g. [98]), some of which are autochthonous to the deep-sea and others that may have sank from shallower depths.

## Conclusions

Hadal microbial communities have been proposed to contain distinct taxa adapted to the unique *in situ* conditions found in trenches. Here, we show that hadal communities within the Kermadec and Mariana trenches are indeed distinct from the abyssal assemblages above them. Hadal communities are enriched in certain taxa that may represent bathytypes, including clades such as the *Marinimicrobia* and specific genera such as *Aquibacter*. Sequences related to known piezophiles were identified in both trenches, albeit in higher abundances in the Kermadec Trench, but at <1% of total communities. These findings suggest similar hadal-associated taxa are present in multiple trenches, potentially transported by deep-ocean currents. Such lineages may be responsible for the higher rates of activity under *in situ* rather than atmospheric pressures determined here. Communities were also distinct between the Mariana and Kermadec trenches, showing varying abundances of cosmopolitan taxa and the presence of unique but rare OTUs. Inter-trench variation was largely driven by differentially abundant heterotrophic *Gammaproteobacteria* that show a remarkable ability to survive long-term pressurization and may be from bathyal and shallower depths where they colonize particles and sink. Trenches are therefore home to unique microbial communities, comprised of autochthonous, pressure-adapted members and ubiquitous genera found throughout the water column.

## Supporting information

S1 Text Supplementary information and methods. (DOCX)

S1 Fig Cross-section by depth and latitude of water sample collection locations in the Mariana and Kermadec trenches. Circles, CTD cast; Triangles, lander.
(TIF)

S2 Fig General nutrient data and cell counts within seawater from the Mariana and Kermadec trenches. Red circles, Kermadec Trench; Blue circles, Mariana Trench.
(TIF)

S3 Fig The percentage of active cells at either (A) atmospheric or (B) *in situ* pressures, where filled circles were obtained at <13°C (excluding the surface sample) and open circles at >15°C. C; The percentage of active cells of a Mariana Trench surface water sample as a function of temperature and pressure.
(TIF)

S4 Fig A; Beta diversity community comparisons between water samples visualized by either weighted Unifrac or Bray-Curtis ordinations. B; Heirarchical clustering of samples based on Bray-Curtis dissimilarity colored broadly by pelagic collection location.
(TIF)

S5 Fig Phyla showing enrichment within either the hadal or abyssal zones in the Kermadec and Mariana trenches. Positive, hadal; negative, abyssal.
(TIF)

S6 Fig Phylogenetic trees showing the relationship of hadal-enriched OTUs (blue) with other closely related sequences. A, *Aquibacter*; B, *Defluviicoccus*; C, Rhodobacteraceae; D, *Planctomyces*.
(TIF)

S7 Fig Alpha diversity comparisons of the Mariana and Kermadec trenches separated by size fraction. Red, Kermadec Trench; Blue, Mariana Trench.
(TIF)

S8 Fig A; Relative abundances of the nitrogen-cycling bacteria *Nitrosomonas*, *Nitrospina*, and *Nitrospira* in the community Itag data. B; Nitrite concentrations within the Kermadec and Mariana Trenches. C; Relative abundances of *Nitrosomonas* and *Nitrospira* within each abyssal or hadal sampling site in either the Mariana (left) or Kermadec (right) trench plotted as a function of nitrite concentrations.
(TIF)

S9 Fig OTUs showing enrichment within either the >3.0 or 3.0–0.2 μm size fractions within the abyssal and hadal zones. Taxonomic labels represent the lowest discernible taxonomic rank. Positive, >3.0 μm; negative, 3.0–0.2 μm.
(TIF)

S10 Fig Phyla showing enrichment within either the 3.0–0.2 μm or 0.2–0.1 μm size fractions within the abyssal and hadal zones. Positive, 0.2–0.1 μm; negative, 3.0–0.2 μm.
(TIF)

S11 Fig Growth curves of nine representative isolates at 15°C or 4°C under atmospheric or high hydrostatic pressure (MPa) conditions. (TIF)

S12 Fig Total and culturable cell counts of nine representative isolates after 30 days at 4°C and varying hydrostatic pressures. T0; counts prior to long-term pressurization.
(TIF)

S13 Fig Phylogenetic trees of OTUs related to *Pseudoalteromonas* (A), *Psychrobacter* (B), and *Halomonas* (C) that were abundant in surface, abyssal, and hadal samples.
(TIF)

S1 Table Sample collection locations used in this study. (XLSX)

S2 Table Samples used for high throughput 16S rRNA gene community composition analyses. (XLSX)

S3 Table OTUs showing enrichment within either the hadal or abyssal zones in the Kermadec and Mariana trenches. Positive log2FoldChange, hadal; negative abyssal.
(XLSX)

S4 Table OTUs showing enrichment within the hadal zones of either the Kermadec or Mariana trenches. Positive log2FoldChange, Mariana; negative Kermadec.
(XLSX)

S5 Table Colony forming units (CFUs) per mL of sample at different sampling locations based on media type and incubation method. (XLSX)

S6 Table List of isolates obtained at atmospheric pressure and 4C on plates and in bulbs. (XLSX)

S7 Table Isolate information used for growth curve and survival analyses. (XLSX)

S8 Table Relative abundances, OTU rank, and culturing ranks of taxa found at >.05% abundance on 3.0 and 0.2 um size fractions at all depths. Relative abundances shown were determined after combining samples by size fraction and depth zone (Surface (1–131 m), abyssal (500- ~6000 m), hadal (~6000–10525 m)).
(XLSX)
