# Supplementary material for: Vertically distinct microbial communities in the Mariana and Kermadec trenches
Source: PLoS One. 2018 Apr 5;13(4):e0195102. doi: 10.1371/journal.pone.0195102 (PMC5886532; doi:10.1371/journal.pone.0195102)
Supplement: S1 Text — (DOCX) [file pone.0195102.s001.docx]

**SUPPLEMENTARY METHODS S1 Text**

**Title:** Vertically distinct microbial communities in the Mariana and Kermadec trenches

**Authors:** Logan M. Peoples^1^, Sierra Donaldson^1^, Oladayo Osuntokun^1^, Qing Xia^1,2^, Alex Nelson^3^, Jessica Blanton^1^, Eric E. Allen^1^, Matthew J. Church^3,4^, and Douglas H. Bartlett^1^*

**Affiliations:**

1. Marine Biology Research Division, Scripps Institution of Oceanography, University of California San Diego, La Jolla, CA, 92093

2. Department of Soil Science, North Carolina State University, Raleigh, NC, 27695

3. Center for Microbial Oceanography: Research and Education, C-MORE Hale, University of Hawaiʻi at Mānoa, Honolulu, HI, 96822

4. Flathead Lake Biological Station, University of Montana, Polson, MT

* To whom correspondence should be addressed: [dbartlett@ucsd.edu](mailto:dbartlett@ucsd.edu)

**SUPPLEMENTARY METHODS**

*Microbial activity*

BONCAT labeling for active cells was done as previously described (Samo *et al*., 2014). Briefly, filters were incubated for 30 minutes in the presence of Click-iT detection buffer with the fluorophore Alexa Fluor 488 (Thermo Fisher Scientific) and counter-stained with DAPI (Vector Laboratories, Burlingame, CA). Images were immediately acquired with a Nikon TE2000-U inverted epifluorescence microscope using the NIS-Elements 3.0 software (Nikon Instruments, Japan) at 350 nm excitation 457 nm emission for DAPI, 490 nm excitation 528 nm emission for incorporated HPG, and 555 nm excitation 617 emission for photopigment autofluresocence. Exposure for HPG incorporation was ~90ms but was adjusted by sample if necessary. Cells were considered active only if they had a blue DAPI signal and a green FITC signal simultaneously. At least 10 images and 100 cells were counted per sample.

HPG concentrations and length of incubations were chosen based on control experiments conducted with *Photobacterium profundum* SS9 (Pressure optimum, 28 MPa) and *Shewanella benthica* KT99 (Pressure optimum, 90 MPa). Both showed elevated levels of HPG incorporation under high hydrostatic pressures (data not shown; SS9 range, 0.1-50 MPa; KT99 range, 27-90 MPa) compared to atmospheric pressure, but at rates slower than those for surface water samples (Samo *et al*., 2014). The incubation conditions used here similar to those needed to identify other deep-ocean taxa (Hatzenpichler *et al*., 2016).

*DNA sequencing and contaminant screening of community data*

Samples were tagged with Illumina barcodes during a secondary PCR procedure with the following reaction: 98°C for 1 min, 8 cycles of 98°C for 10 sec, 60°C for 20 sec, and 72°C for 30 sec, and 72°C for 2 min. Samples were pooled at equimolar concentrations and sent for sequencing on an Illumina Miseq at the Institute for Genomic Medicine Genomics Center (University of California, San Diego, La Jolla, CA). The most abundant OTUs within the extraction negative controls were screened using BLAST to identify taxa indicative of cross contamination between samples and controls. Only genera that were specifically enriched in the extraction blanks and showed high similarity to those associated with humans, purified water, or other known contaminants (Lusk *et al*., 2014; Salter *et al*., 2014) were identified and removed. These genera include *Acinetobacter*, *Stenotrophomonas*, *Methylobacterium*, *Achromobacter*, *Rhizobium*, *Tetragenococcus*, *Escherichia-Shigella*, *Ferribacterium*, *Massilia*, *Curvibacter*, *Ralstonia*, *Variovorax*, *Deinococcus*, *Anoxybacillus*, *Aeribacillus*, *Brevundimonas*, *Enterobacter*, *Streptococcus*, *Burkholderia*, *Staphylococcus*, *Sphingomonas*, *Sphingobium*, *Sphingobacterium*, *Bradyrhizobium*, *Paenibacillus*, and the family Comamonadaceae. *Pseudomonas* OTUs were not removed as a previous study identified this genus as an abundant member of hadopelagic waters in the Mariana Trench (Nunoura *et al*., 2015).

*Media used for isolation of microbes*

Samples from the Kermadec Trench were inoculated into 2216 Marine Medium, A1 medium (5 g potato starch, 2 g yeast extract, 1 g peptone, 0.5 g NH_4_Cl, 0.01 g L-methionine, 500 mL 0.2 μm-filtered autoclaved seawater collected from the Ellen Browning Scripps Pier), or a seawater minimal medium (10 mM NH_4_Cl, 14 mg Na_2_HPO_4_, American Type Culture Collection (ATCC) vitamin supplement (ATCC, Manassas, VA), ATCC trace mineral supplement, 1 L 0.2 μm-filtered Scripps Pier seawater). In contrast, samples from the Mariana Trench were incubated in only 2216. These media types have been previously used on trench samples and have yielded success.

*Nutrient data*

The ammonium values obtained in this study may be anomalously high for pelagic deep-ocean locations. For example, values in the pelagic Puerto Rico and Mariana trenches have found ammonium concentrations of less than 100 nM (Eloe *et al*., 2011b; Nunoura *et al*., 2015). However, benthic boundary layer nutrient concentrations may differ from those in the pelagic water column as a result of sediment resuspension (Pilskaln *et al*., 1998), mixing ammonium-rich sediment pore water (Nunoura *et al*., 2013) with the overlying water column. Such mixing may be exacerbated in deep-ocean trenches because of topographical funneling of organic matter and the potential for earthquake-induced seafloor deformation. In the Ogasawara Trench the ammonium concentration in seawater collected just above the seafloor was reported as 1.5 μM (Nunoura *et al*., 2013), while benthic boundary seawater collected from the Mariana Trench had concentrations greater than 0.15 μM (Tarn *et al*., 2016).

However, the influence of sediment and organic matter resuspension may not be the only reason for the high ammonium concentrations identified here. While freezing is a reliable way to store samples when immediate analysis is not feasible (Dore *et al*., 1996), measurements of ammonium after freezing have shown high variability after long-term storage (Newell, 1967; Morse *et al*., 1982; personal communication, SIO Oceanographic Data Facility). Because the samples in this study were frozen shipboard prior to analysis on land, we urge caution in interpreting the ammonium values presented here.

**ADDITIONAL SUPPLEMENTARY REFERENCES**

Dore JE, Houlihan T, Hebel DV, Tien G, Tupas L, Karl DM. (1996). Freezing as a method of sample preservation for the analysis of dissolved inorganic nutrients in seawater. *Marine Chemistry* **53**: 173-185.

Lusk RW. (2014). Diverse and widespread contamination evident in the unmapped depths of high throughput sequencing data. *PLoS ONE* **9**: e110808.

Morse JW, Hunt M, Zullig J, Mucci A, Mendez T. (1982). A comparison of techniques for preserving dissolved nutrients in open ocean seawater samples. *Ocean Science and Engineering* **7**: 75-106.

Newell BS. (1967). The determination of ammonia in sea water. *J. Mar. Biol. Ass. U.K.* **47**: 271-280.

Pilskaln CH, Churchill JH, Mayer LM. (1998). Resuspension of sediment by bottom trawling in the Gulf of Maine and potential geochemical consequences. *Conservation Biology* **12**: 1223-1229.

Salter SJ, Cox MJ, Turek EM, Calus ST, Cookson WO, Moffatt MF, *et al*. (2014). Reagent and laboratory contamination can critically impact sequence-based microbiome analyses. *BMC Biology* **12**: 87.
