# Supplementary figures and images for: Vertically distinct microbial communities in the Mariana and Kermadec trenches
Source: PLoS One. 2018 Apr 5;13(4):e0195102. doi: 10.1371/journal.pone.0195102 (PMC5886532; doi:10.1371/journal.pone.0195102)

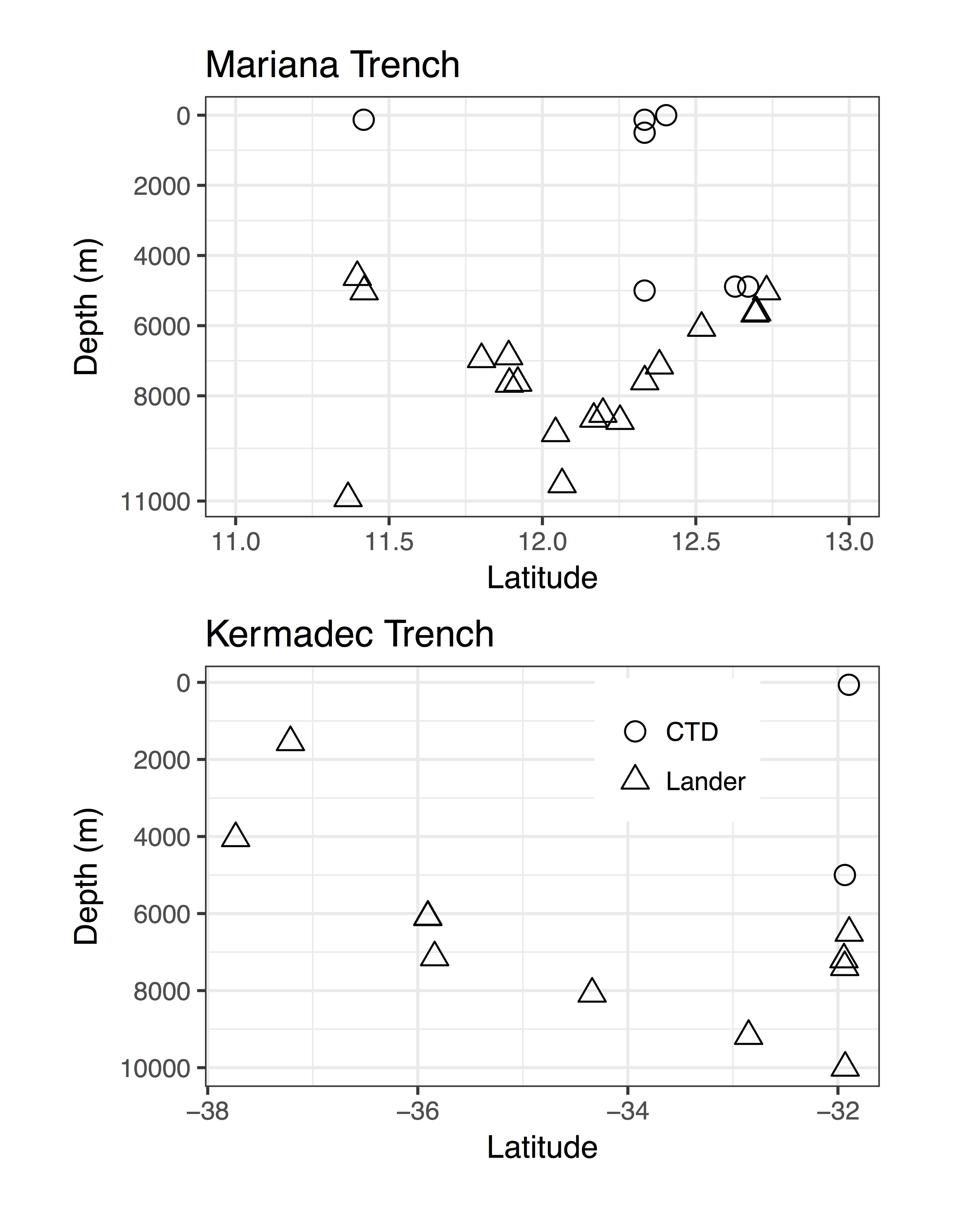

Supplement: S1 Fig — Circles, CTD cast; Triangles, lander. (TIF) [file pone.0195102.s002.tif]

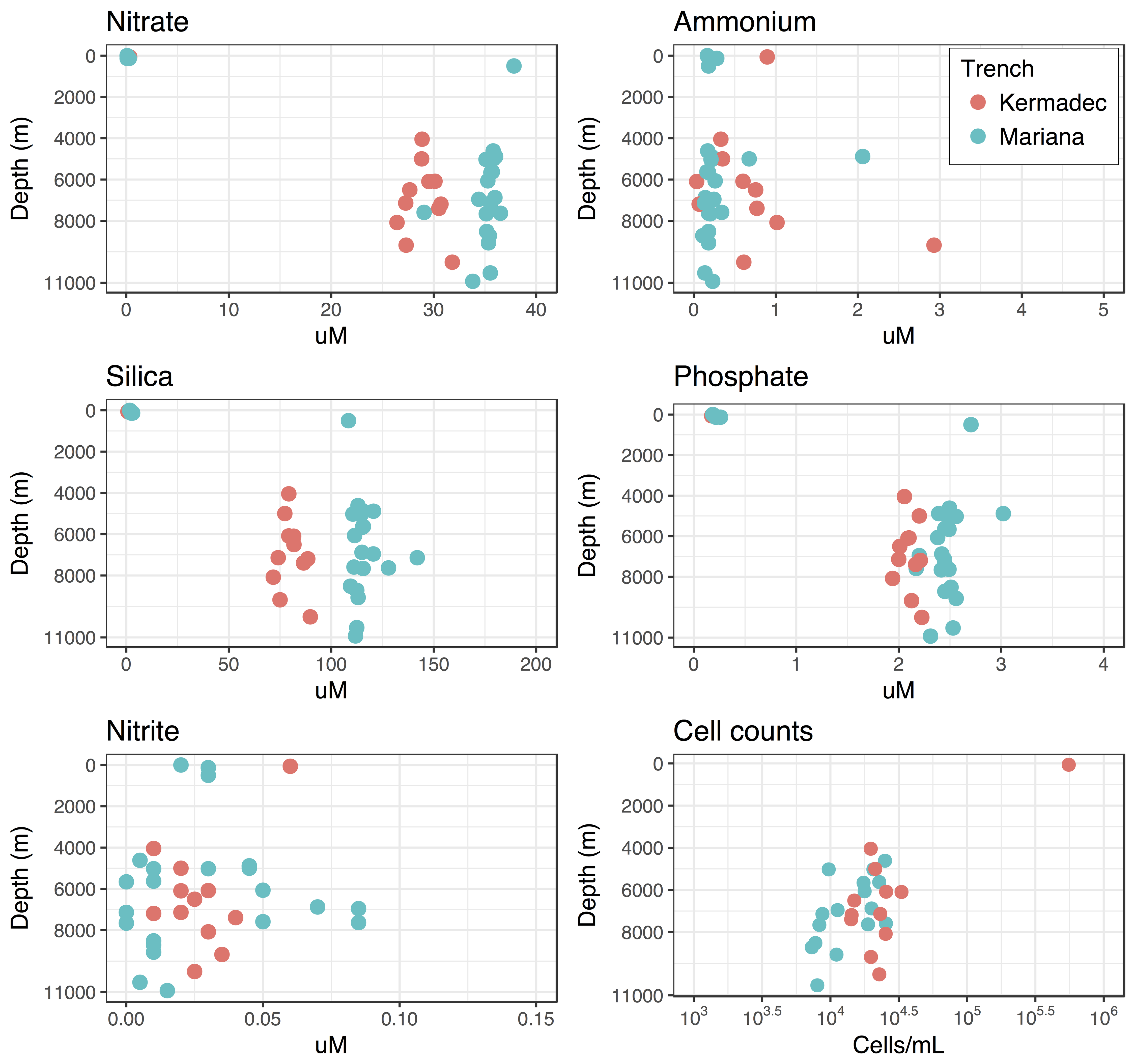

Supplement: S2 Fig — Red circles, Kermadec Trench; Blue circles, Mariana Trench. (TIF) [file pone.0195102.s003.tif]

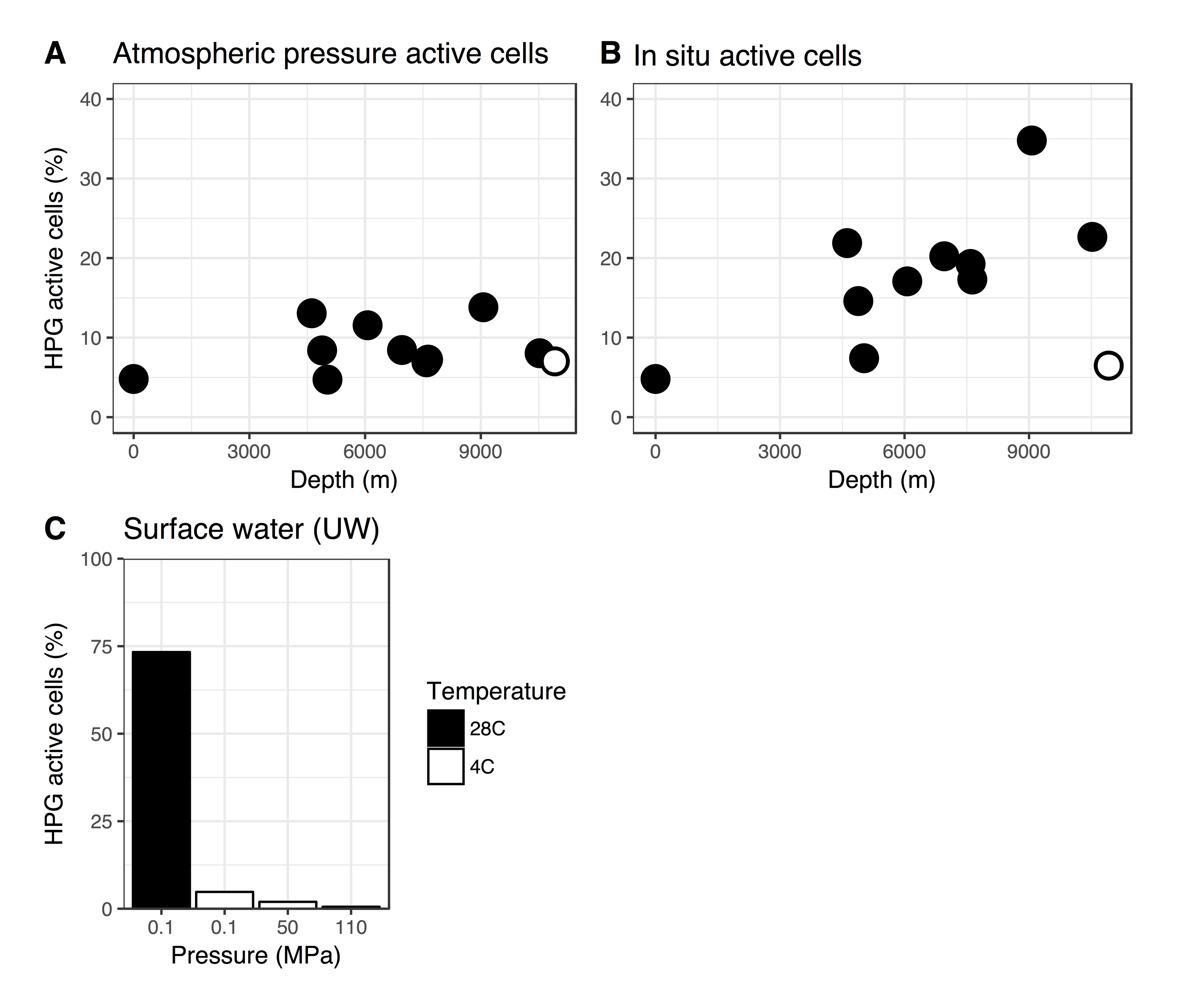

Supplement: S3 Fig — The percentage of active cells at either (A) atmospheric or (B) in situ pressures, where filled circles were obtained at <13°C (excluding the surface sample) and open circles at >15°C. C; The percentage of active cells of a Mariana Trench surface water sample as a function of temperature and pressure. (TIF) [file pone.0195102.s004.tif]

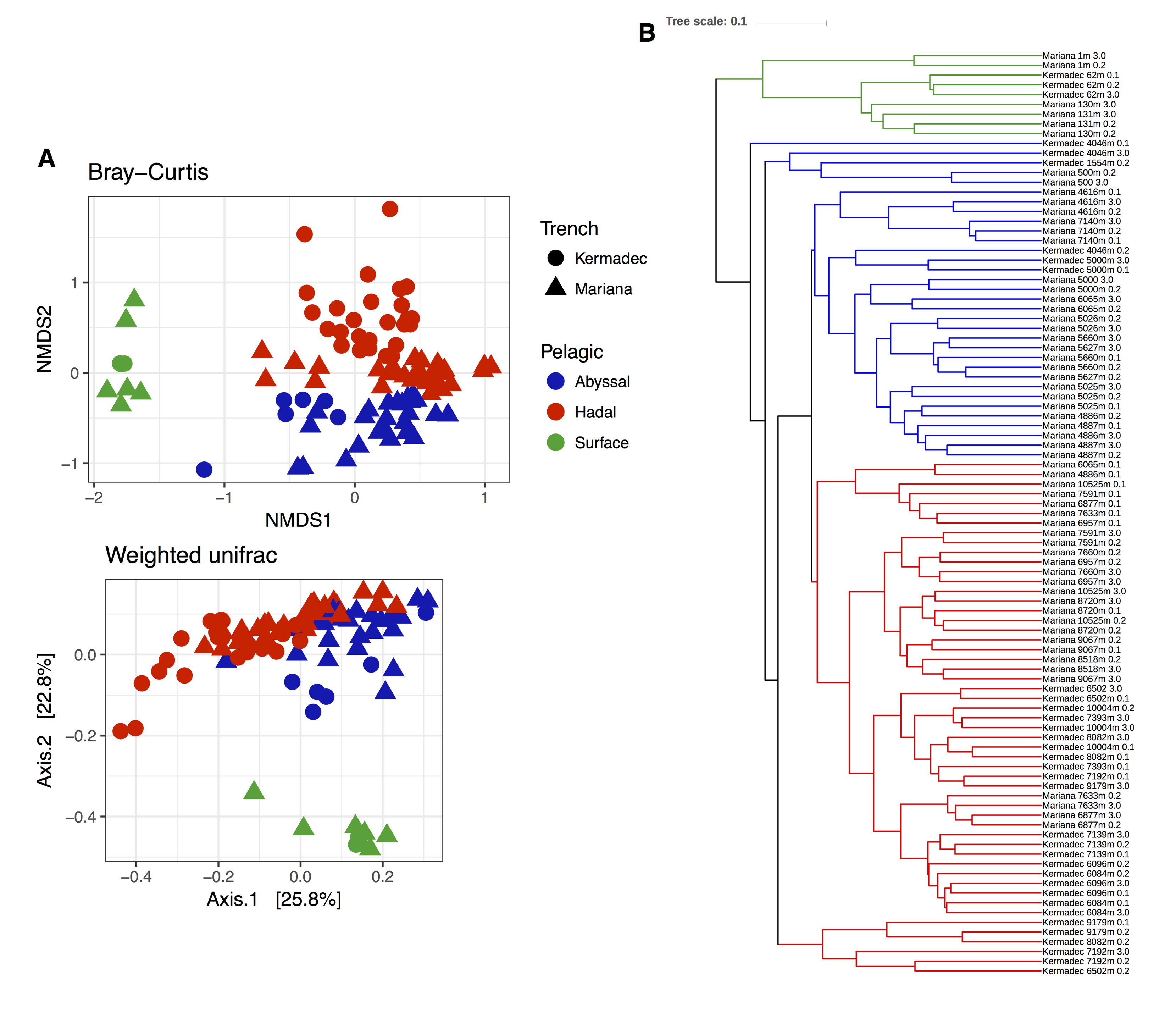

Supplement: S4 Fig — A; Beta diversity community comparisons between water samples visualized by either weighted Unifrac or Bray-Curtis ordinations. B; Heirarchical clustering of samples based on Bray-Curtis dissimilarity colored broadly by pelagic collection location. (TIF) [file pone.0195102.s005.tif]

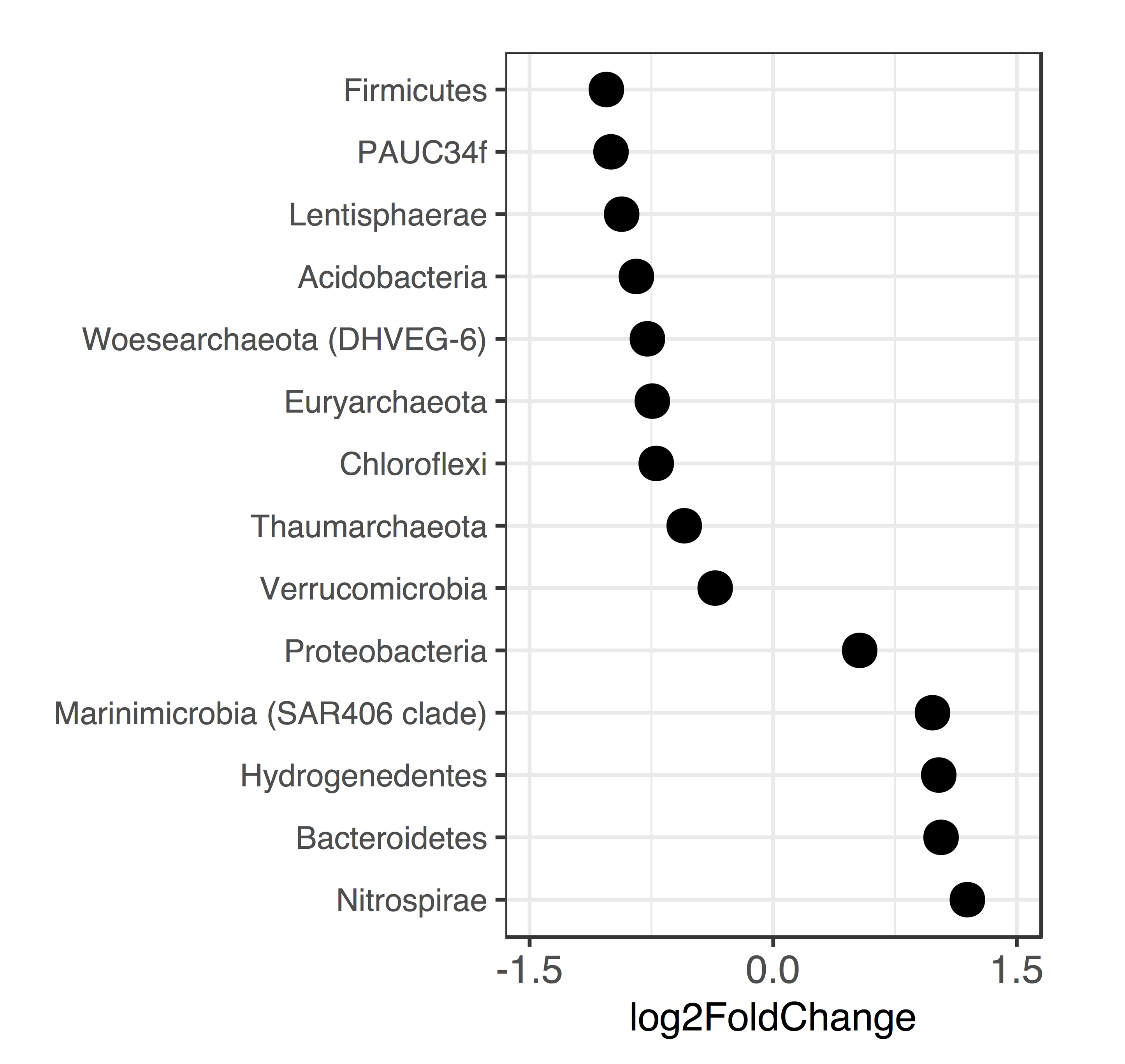

Supplement: S5 Fig — Positive, hadal; negative, abyssal. (TIF) [file pone.0195102.s006.tif]

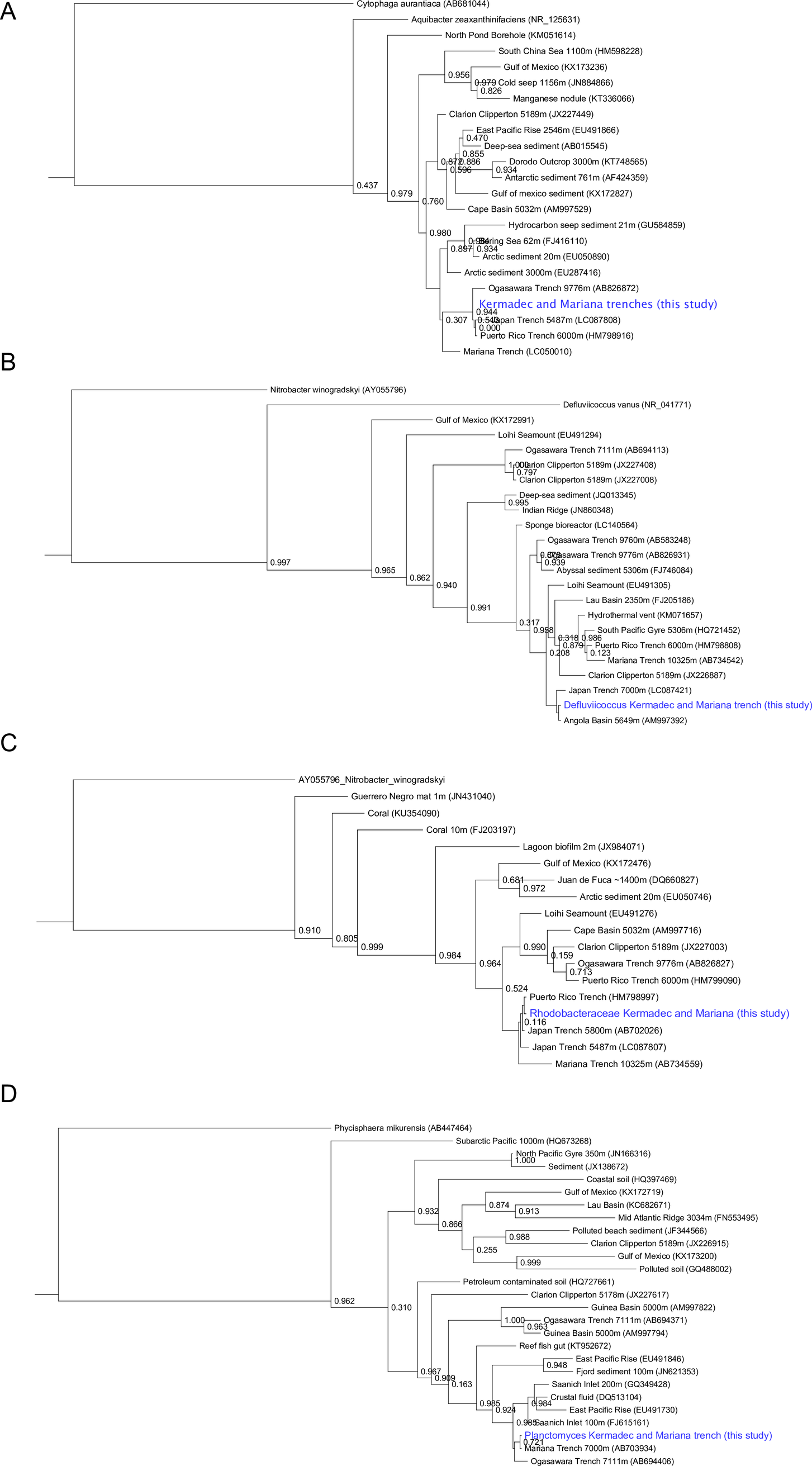

Supplement: S6 Fig — A, Aquibacter; B, Defluviicoccus; C, Rhodobacteraceae; D, Planctomyces. (TIF) [file pone.0195102.s007.tif]

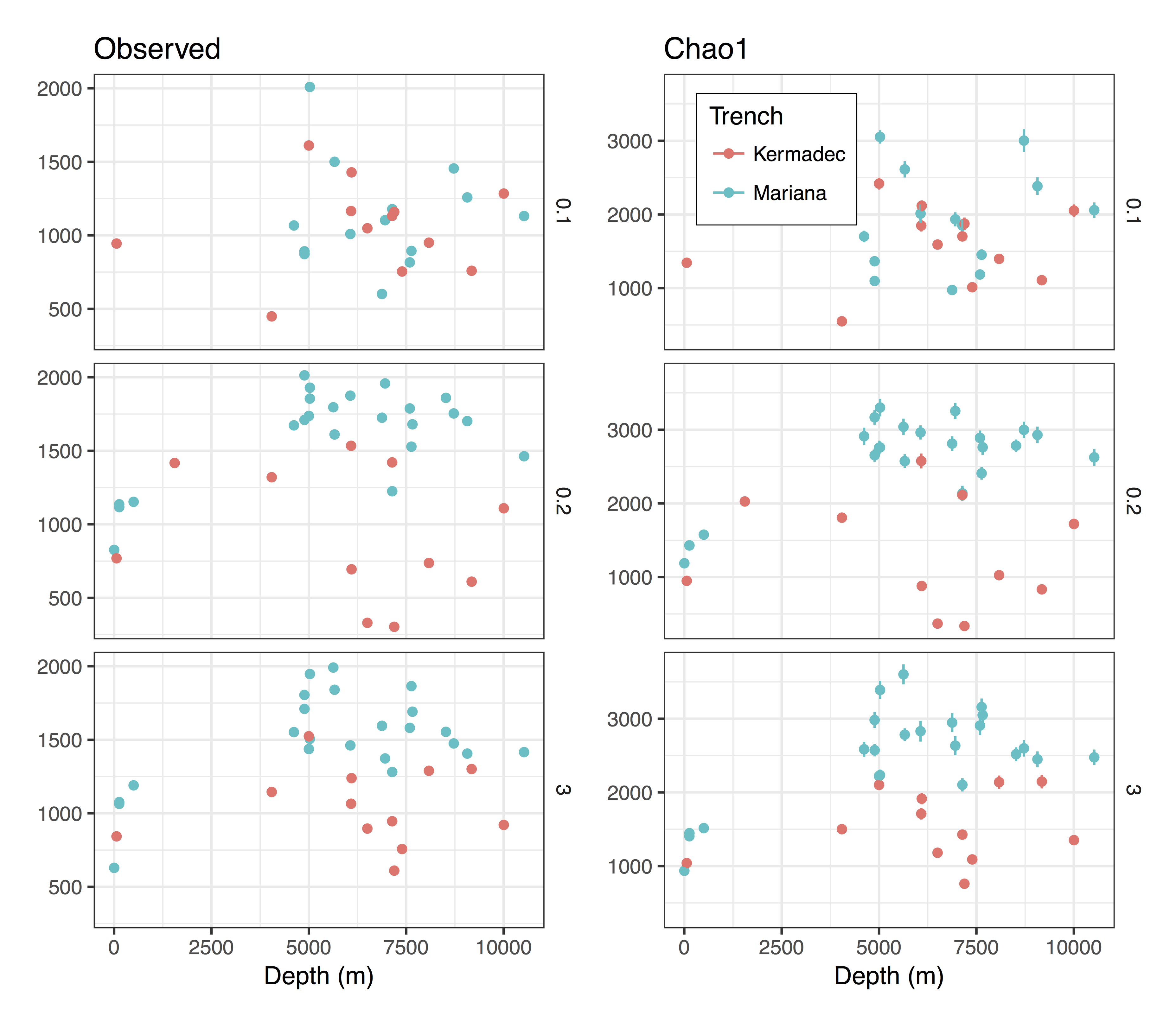

Supplement: S7 Fig — Red, Kermadec Trench; Blue, Mariana Trench. (TIF) [file pone.0195102.s008.tif]

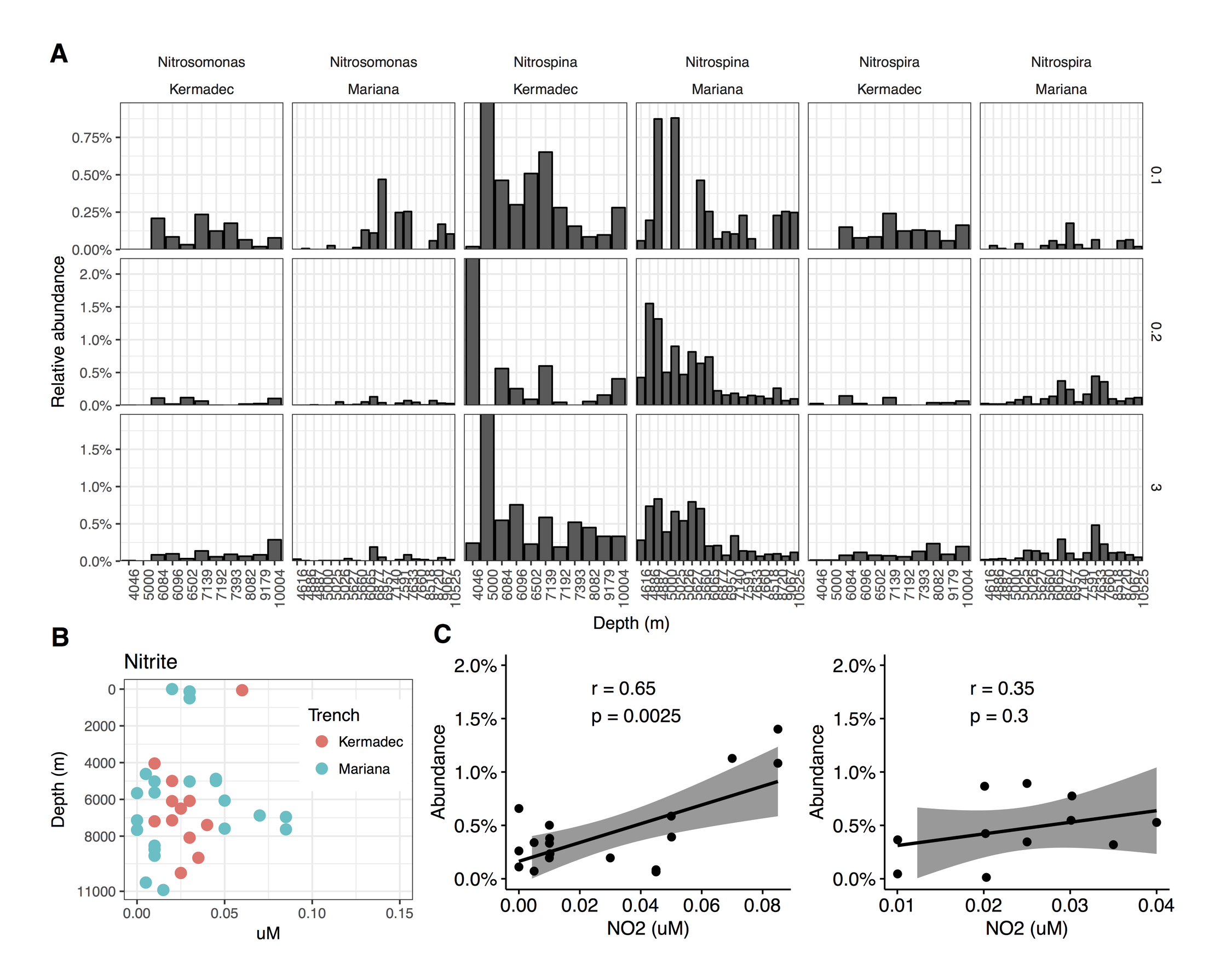

Supplement: S8 Fig — A; Relative abundances of the nitrogen-cycling bacteria Nitrosomonas, Nitrospina, and Nitrospira in the community Itag data. B; Nitrite concentrations within the Kermadec and Mariana Trenches. C; Relative abundances of Nitrosomonas and Nitrospira within each abyssal or hadal sampling site in either the Mariana (left) or Kermadec (right) trench plotted as a function of nitrite concentrations. (TIF) [file pone.0195102.s009.tif]

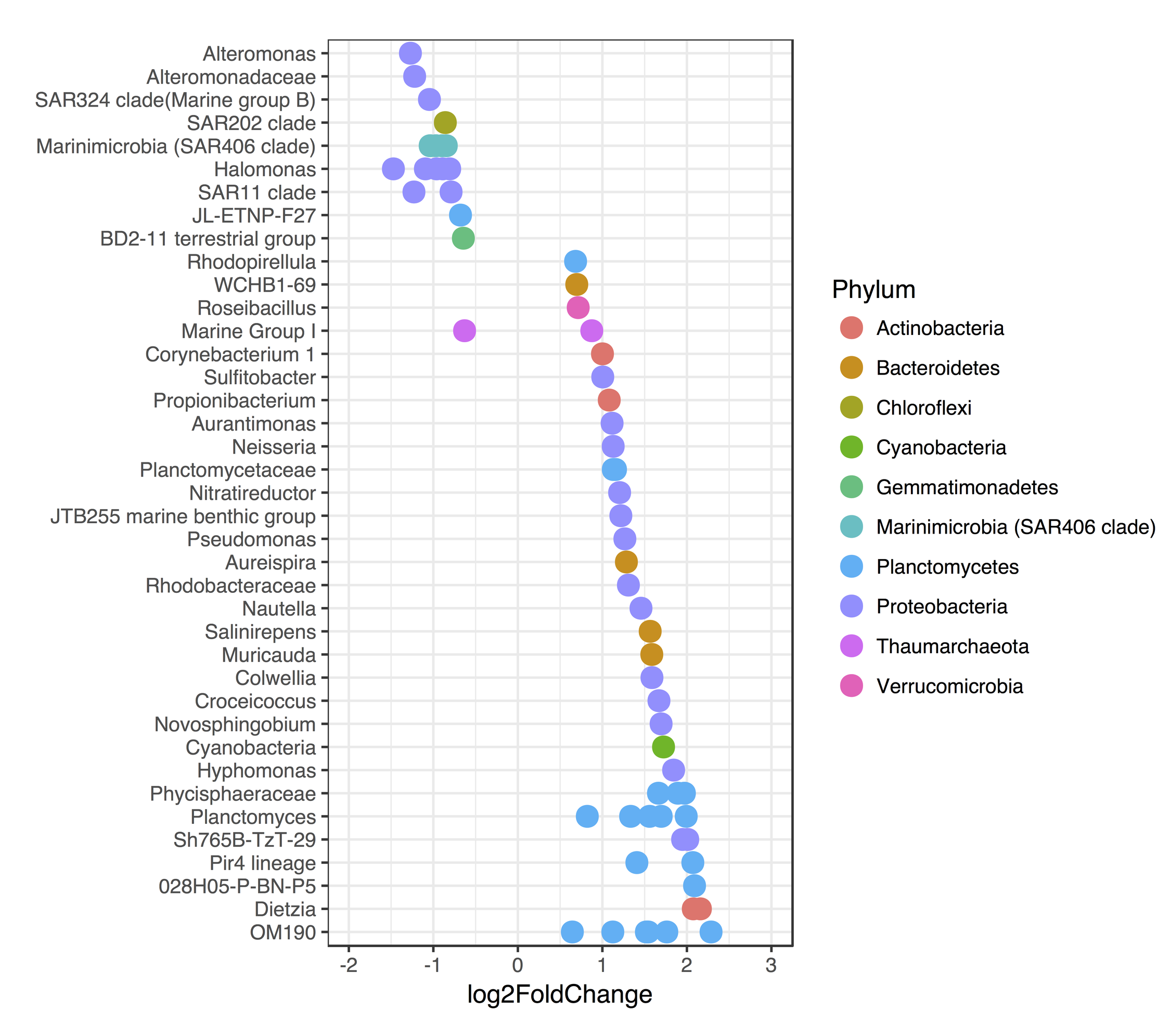

Supplement: S9 Fig — Taxonomic labels represent the lowest discernible taxonomic rank. Positive, >3.0 μm; negative, 3.0–0.2 μm. (TIF) [file pone.0195102.s010.tif]

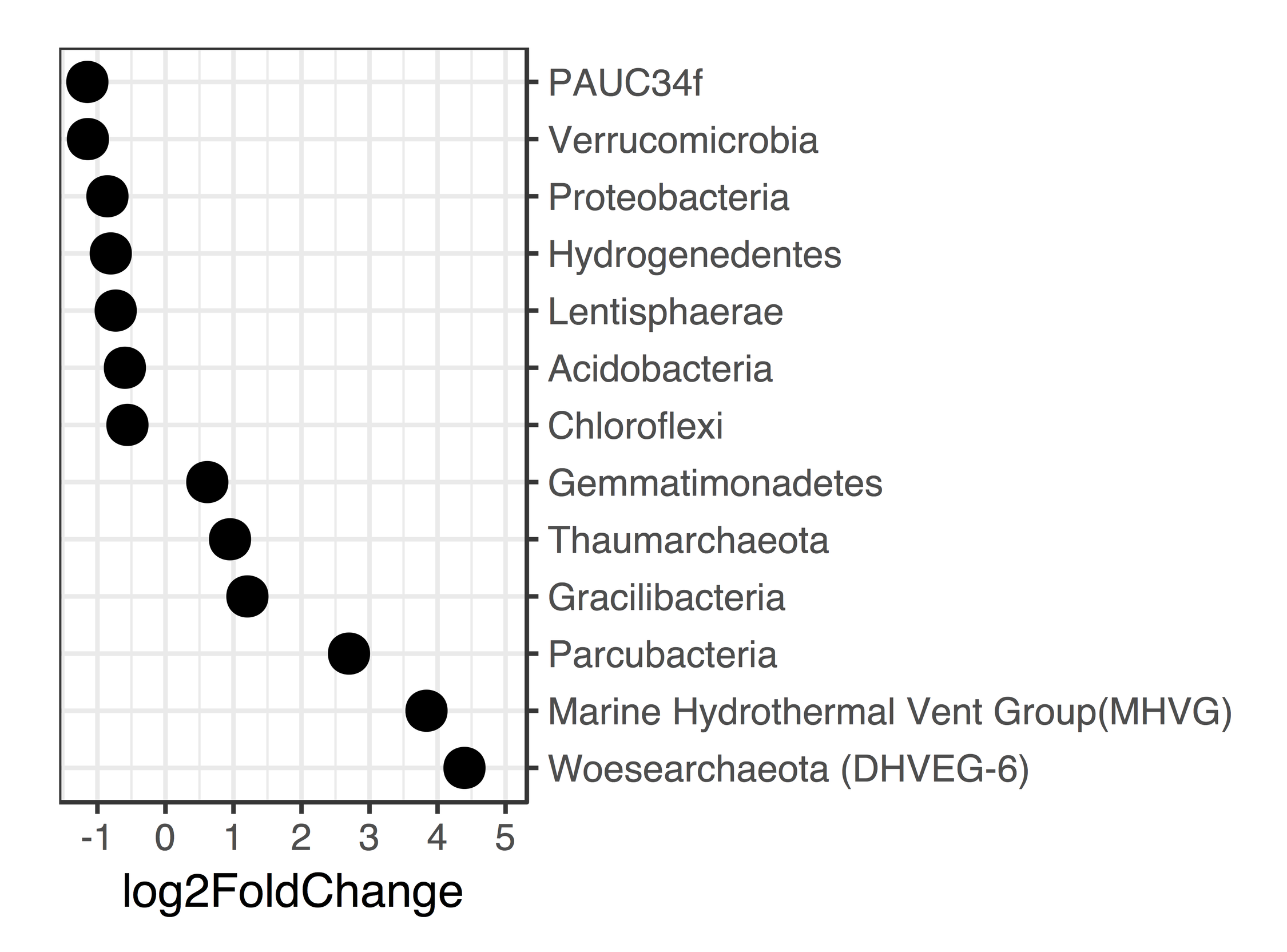

Supplement: S10 Fig — Positive, 0.2–0.1 μm; negative, 3.0–0.2 μm. (TIF) [file pone.0195102.s011.tif]

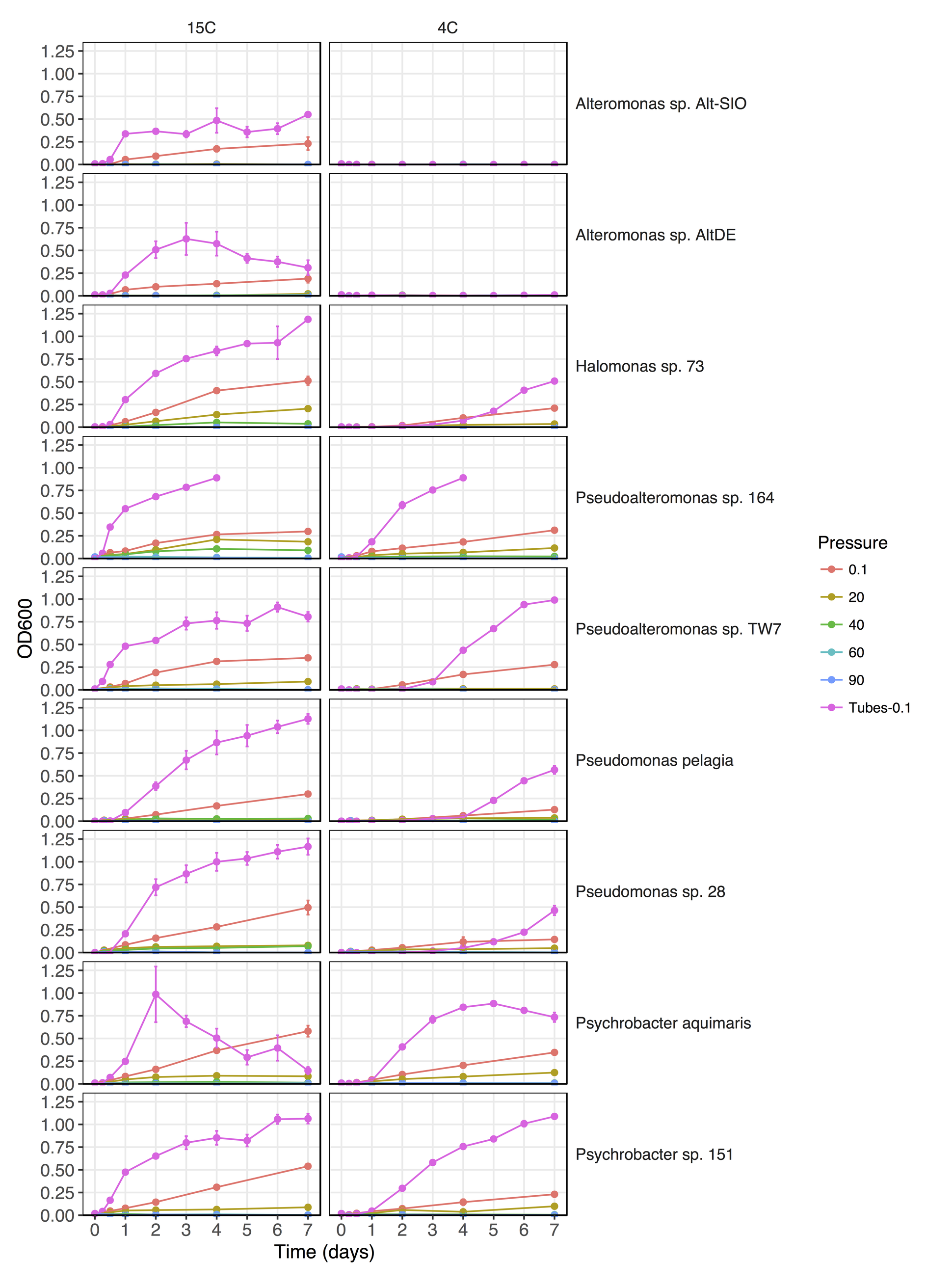

Supplement: S11 Fig — (TIF) [file pone.0195102.s012.tif]

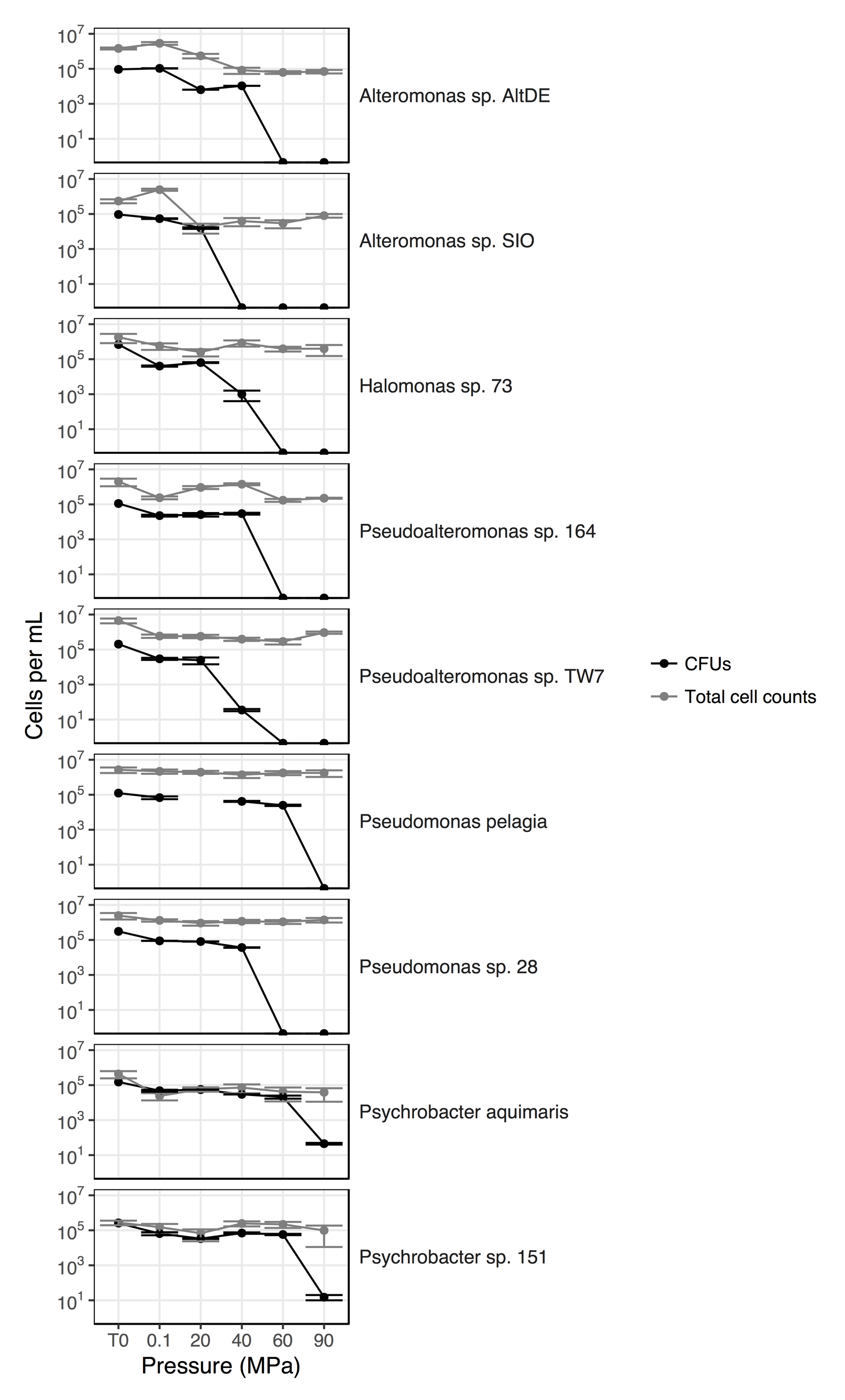

Supplement: S12 Fig — T0; counts prior to long-term pressurization. (TIF) [file pone.0195102.s013.tif]

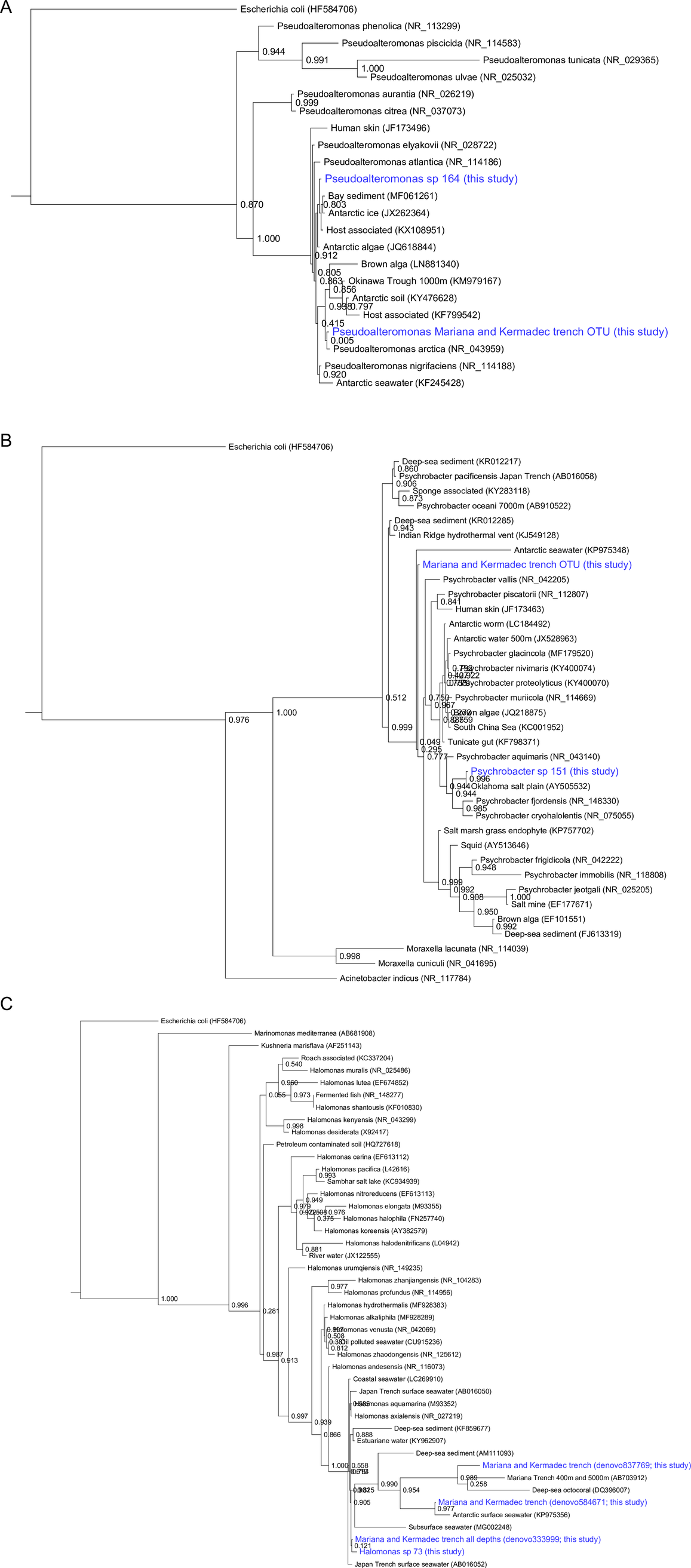

Supplement: S13 Fig — Phylogenetic trees of OTUs related to Pseudoalteromonas (A), Psychrobacter (B), and Halomonas (C) that were abundant in surface, abyssal, and hadal samples. (TIF) [file pone.0195102.s014.tif]
